# Platelet–Monocyte Aggregate Instigates Inflammation and Vasculopathy in Kawasaki Disease

**DOI:** 10.1002/advs.202406282

**Published:** 2024-12-12

**Authors:** Yuan Zhang, Cuiping Jia, Manli Guo, Qian Chen, Ying Wen, Ting Wang, Yinyin Xie, Xuejiao Fan, Jingwen Gao, Timur O. Yarovinsky, Renjing Liu, Zhiyong Jiang, Mengmeng Wang, Jin Zhou, Di Che, Lanyan Fu, Richard Edelson, Xiaoqiong Gu, John Hwa, Wai Ho Tang

**Affiliations:** ^1^ Institute of Pediatrics Guangzhou Women and Children's Medical Center Guangzhou Medical University Guangzhou 510623 China; ^2^ Yale Cardiovascular Research Center Section of Cardiovascular Medicine Department of Internal Medicine Yale University School of Medicine New Haven CT 06511 USA; ^3^ Victor Chang Cardiac Research Institute Sydney 2010 Australia; ^4^ Department of Blood Transfusion Guangzhou Women and Children's Medical Center Guangzhou Medical University Guangzhou 510623 China; ^5^ Department of Children's Ophtalmology Guangzhou Women and Children's Medical Center Guangzhou Medical University Guangzhou 510623 China; ^6^ Department of Biological Specimen Bank Guangzhou Women and Children's Medical Center Guangzhou Medical University Guangzhou 510623 China; ^7^ Department of Dermatology School of Medicine Yale University New Haven CT 06511 USA; ^8^ School of Nursing and Health Studies Hong Kong Metropolitan University Kowloon Hong Kong SAR China

**Keywords:** inflammation, Kawasaki disease, monocyte, platelet, vasculopathy

## Abstract

Kawasaki disease (KD) is a severe acute febrile illness and systemic vasculitis that causes coronary artery aneurysms in young children. Platelet hyperreactivity and an aberrant immune response are key indicators of KD; however, the mechanism by which hyperactive platelets contribute to inflammation and vasculopathy in KD remains unclear. A cytokine‐mediated positive feedback loop between KD platelets and monocytes is identified. KD platelet–monocyte aggregates (MPAs) are mediated by an initial interaction of P‐selectin (cluster of differentiation 62P, CD62p) and its glycoprotein ligand 1 (PSGL‐1). This is followed by a coordinated interaction of platelet glycoprotein (GP)Ibα with monocyte CD11b. Monocyte‐activated platelets initiate transforming growth factor (TGF)β1 release, which results in nuclear localization of nuclear factor kappaB in monocytes, therefore, driving the phenotypic conversion of classical monocytes (CD14^+^CD16^−^) into proinflammatory monocytes (CD14^+^CD16^+^). The platelet‐activated monocytes release interleukin‐1 and tissue necrotic factor‐α, which promote further platelet activation. KD‐induced inflammation and vasculopathy are prevented by inhibiting the components of this positive feedback loop. Notably, mice deficient in platelet TGFβ1 show less MPA and CD14^+^CD16^+^ monocytes, along with reduced inflammation and vasculopathy. These findings reveal that platelet–monocyte interactive proteins (CD62p/PSGL‐1 and (GP)Ibα/CD11b) and cytokine mediators (platelet TGFβ1) are potential biomarkers and therapeutic targets for KD vasculopathy.

## Graphical abstract

The formation of monocyte–platelet aggregate (MPA) induces the phenotypic conversion of monocytes into proinflammatory monocytes via the platelet release of transforming growth factor (TGF)β1, resulting in inflammation (interleukin‐1β, tissue necrotic factor‐α) and vasculopathy in Kawasaki disease (KD). Blocking MPA formation or reducing platelet TGFβ1 may provide potential biomarkers and therapeutic targets for KD vasculopathy.

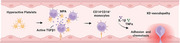

## 1 Introduction

Kawasaki disease (KD) is a systemic vasculitis that predominantly affects children <5 years old.^[^ ^1^ ^]^ Conventionally, it affects medium‐sized arteries, particularly coronary arteries.^[^ ^2^ ^]^ Notably, platelet hyperreactivity^[^ ^3^ ^]^ and inflammation^[^ ^4^ ^]^ are the key indicators of KD, and standard treatment requires combining oral aspirin and high‐dose intravenous immunoglobulin (IVIG).^[^ ^4^ ^]^ However, some children with KD develop coronary artery aneurysm (CAA), and young adults experience adverse cardiac events even after receiving the standard therapy,^[^ ^5^ ^]^ which lead to significant morbidity and mortality. Therefore, there is an urgent need to understand the mechanism of KD‐induced vasculopathy and develop precise mechanism‐based therapies.

Notably, monocytes and their derived macrophages are important innate effectors in the pathogenesis of various inflammatory diseases. Human monocytes comprise three main subsets based on the expression of cluster of differentiation 14 (*CD14)*, the lipopolysaccharide (LPS) coreceptor) and CD16 (Fc gamma receptor III, FcγRIII). These subsets include the classical (CD14^+^CD16^−^), the intermediate (CD14^+^CD16^+^), and the nonclassical (CD14^low^CD16^+^) monocytes.^[^ ^6^ ^]^ The intermediate monocyte is a recently identified subtype that has the capacity to produce proinflammatory cytokines such as tissue necrotic factor (TNF)‐α, interleukin (IL)‐1β, and IL‐6.^[^ ^7^ ^]^ Furthermore, expansion of intermediate monocytes has been reported in atherosclerosis,^[^ ^8^ ^]^ ischemic reperfusion injury,^[^ ^9^ ^]^ rheumatoid arthritis,^[^ ^10^ ^]^ and Crohn's disease.^[^ ^11^ ^]^ Additionally, the ratio of intermediate monocytes in circulation and its correlation with disease severity has been identified.^[^ ^12^ ^]^ However, the factors leading to conversion to intermediate monocytes remain unclear.

Moreover, platelets are immune effector cells that function spanning from acute inflammatory response to adaptive immunity.^[^ ^13^ ^]^ Their immune response includes the release of adhesion molecules, chemokines, and cytokines, which are essential for recruiting neutrophils and monocytes. The recruited immune cells respond critically in acute inflammation.^[^ ^14^ ^]^ Furthermore, platelets interact with leukocytes via toll‐like receptors, complement receptors, and integrins, which leads to platelet–leucocyte aggregate formation.^[^ ^14^ ^]^ Reportedly, platelet–monocyte aggregate (MPA) is increased in patients with KD^[^ ^15^ ^]^ and in mice injected with *Lactobacillus casei* cell wall extract (LCWE),^[^ ^16^ ^]^ which is a well‐established murine model of KD. However, the role of MPA in KD vasculopathy remains unclear.

In this study, using KD patient samples and murine model, we found that MPA instigates inflammation and vasculopathy during acute KD, targeting the monocyte–platelet aggregation restores monocyte homeostasis, and improves the vasculopathy in LCWE‐induced KD murine model, suggesting a novel therapeutic target in KD vasculopathy.

## 2 Results

### 2.1 The Formation of Platelet–Monocyte Aggregate Is Associated with Coronary Pathology and Serves as a Biomarker for Coronary Artery Aneurysm

**Figure 1:**
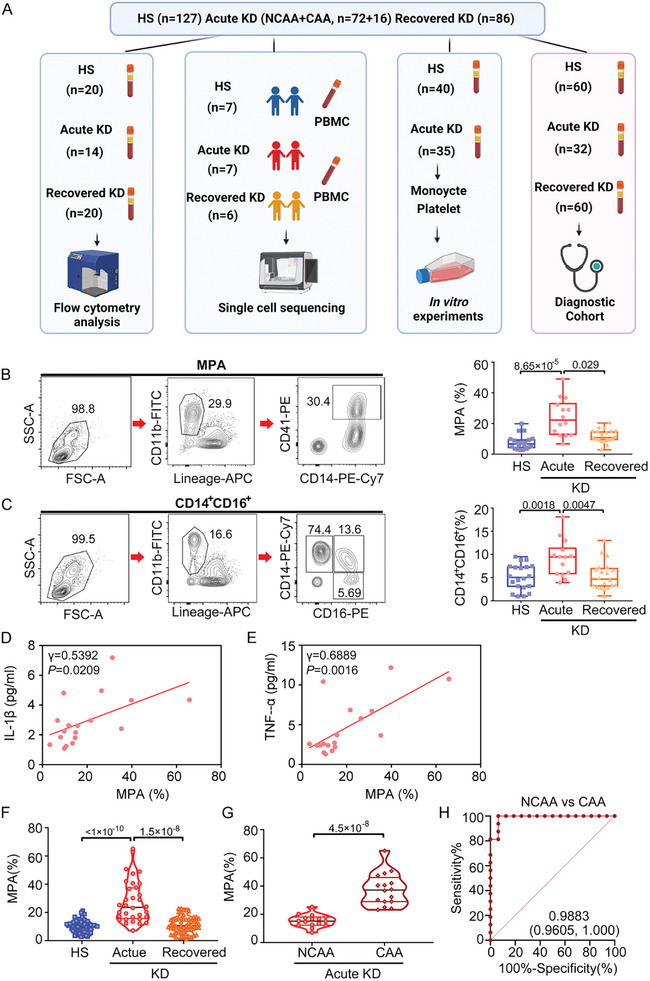
MPA is associated with coronary pathology and serves as a biomarker for coronary artery aneurysm. A) Flowchart depicting the overall experimental design of this study. B) Gating strategy for MPA (CD14^+^CD41^+^) and box plots showing the cell proportion of MPA (CD14^+^CD41^+^) in PBMC from participants. Lineage‐APC indicates anti‐human Lineage Cocktail (CD3, CD19, CD20, CD56). Kruskal–Wallis test and Dunn's multiple comparisons test. C) Gating strategy for CD14^+^CD16^+^ monocytes and box plots showing the cell proportion of CD14^+^CD16^+^ monocytes in PBMC from participants. Lineage‐APC indicates anti‐human Lineage Cocktail (CD3, CD19, CD20, CD56). One‐way ANOVA and Tukey's multiple comparisons test. D,E) Correlation of MPA ratio to (D) IL‐1β (*n* = 18), (E) TNF‐ɑ (*n* = 18) in individuals with Acute KD. F) The proportion (%) of MPA in HS (*n* = 60), Acute KD (*n* = 32), and Recovered KD (*n* = 60) in the diagnostic cohort was measured relative to Lin^−^ (CD3, CD19, CD20, CD56) CD11b^+^ cells. Kruskal–Wallis test and Dunn's multiple comparisons test. G) The proportion (%) of MPA in patients with NCAA (*n* = 16) and CAA (*n* = 16) during Acute KD was measured relative to Lin^−^ (CD3, CD19, CD20, CD56) CD11b^+^ cells. Unpaired *t* test. H) A receiver operating characteristic (ROC) analysis was performed to evaluate the ability of the MPA ratio during the acute phase to distinguish KD patients with NCAA from CAA. HS, healthy subject; KD, Kawasaki disease; MPA, platelet–monocyte aggregate; NCAA, noncoronary artery aneurysm; CAA, coronary artery aneurysm; ANOVA, analysis of variance.

In our prospective cohort involving children, we collected peripheral blood mononuclear cells (PBMCs) from 174 individuals with KD (acute KD, *n* = 88; recovered KD, *n* = 86) and 127 healthy subjects (HSs). The samples collected were used for flow cytometry analysis, single‐cell sequencing, in vitro experiments, and assessment for potential biomarkers for CAA development (**Figure** **1**A and **Table** **1**). The MPA subset, defined as CD14^+^CD41^+^ monocytes, was determined using flow cytometry analysis, as shown in the density plot (Figure 1B). Additionally, the proportion (%) of MPA was measured relative to Lin^−^ (CD3, CD19, CD20, CD56) CD11b^+^ cells. Furthermore, acute KD showed a significant increase in MPA (24.00 ± 12.15%, *p* = 8.65 × 10^−6^) when compared with the HS (7.685 ± 4.664%). However, the increase was attenuated in recovered KD (11.67 ± 4.258%, *p* = 0.029) (Figure 1B). The CD14^+^CD16^+^ monocytes showed a similar trend (9.169 ± 3.991% in acute KD vs 5.083 ± 2.777% in HS, *p* = 0.0018; vs 5.444 ± 3.045% in recovered KD, *p* = 0.0047) (Figure 1C). In addition, MPA (%) was positively correlated with plasma levels of IL‐1β (*p* = 0.0209) (Figure 1D) and TNF‐α (*p* = 0.0016) (Figure 1E), with both suggested as requirements for inducing KD vasculopathy.^[^ ^17^ ^]^ In our second cohort involving randomly enrolled participants, MPA levels were measured at hospital admission before classifying CAA or non‐CAA (NCAA) to assess the potential of MPA for predicting CAA development. The results showed that the percentage of MPA was significantly higher in patients with acute KD (Figure 1F) with a median interquartile range (IQR) level of 23.65 [15.71–37.40], than in those with recovered KD (10.65 [6.723–14.98]) and HS (10.00 [7.010–11.93]), respectively. Furthermore, the proportion of MPA in patients with CAA was significantly higher than in those with NCAA during acute KD (*p* = 4.5 × 10^−8^) (Figure 1G). MPA may serve as a potential marker for predicting CAA development with an area under the curve of 0.9883 [95% confidence interval (CI) 0.9605–1.000] (Figure 1H).

**Table 1:** Demographics, characteristics of participants recruited in our study. *p*‐value was calculated by Kruskal–Wallis test followed by Dunn's multiple comparisons test for continuous variables. Mann–Whitney *U* test was used to analyze differences between NCAA and CAA.

Variables	HS	Acute KD	Recovered KD	*p*‐value
	*n* = 127	*n* = 88	*n* = 86	
Age [months] (IQR)	31 (17–43)	23 (13–50)	34.5 (14–48)	NS
Male, *n* [%]	93 (73.2)	60 (68.2)	63 (73.3)	NS
Coronary artery aneurysm, *n* [%]				
SCAA	NA	11 (12.5)	8 (9.3)	
MCAA	NA	4 (4.5)	9 (10.5)	
GCAA	NA	1 (1.1)	6 (7)	
Normal	127 (100)	72 (81.8)	63 (73.3)	
Fever time [days]	NA	5 (4–7)	5 (4–6)	NS
Laboratory data, median (IQR)				
PLT [10^9^ L^−1^]	323 (281–377)	371 (314–441)***	348 (291.5–404.3)	0.00067
MPV [fL]	9.6 (9.15–10.25)	9.5 (9.1–10.3)	9.2 (8.9–9.8)**	0.004
PDW [%]	10 (9.1–11.4)	9.8 (9.0–11.1)	9.5 (8.8–10.6)*	0.038
WBC [10^9^ L^−1^]	8.1 (6.6–9.5)	13.3 (9.8–17.6)****	7.3 (6.7–8.6)^####^	<0.0001 <0.0001
Mono [10^9^ L^−1^]	0.42 (0.35–0.52)	0.99 (0.61–1.29)****	0.52 (0.39–0.64)*^####^	<0.0001 0.0185
Mono [%]	5.1 (4.9–6.0)	7.1 (5.0–9.0)****	6.7 (5.7–8.5)****	<0.0001 <0.0001
HGB [g L^−1^]	122 (116–128)	109 (101–114)****	123 (115–127)^####^	<0.0001 <0.0001
CRP [mg L^−1^]	NA	61.6 (33.1–95.9)	0.65 (0.5–1.2)^####^	<0.0001
ESR [mm h^−1^]	NA	54 (31.3–71.8)	10 (8–15.8)^####^	<0.0001

Abbreviations: HS, healthy subject; KD, Kawasaki disease; coronary artery aneurysm is determined by the score of coronary angiography, expressed as *Z*‐worst, which is the inner diameter of the proximal coronary artery segment/body surface area. A normal coronary artery dimension was defined as a *Z*‐worst <2.5. Small coronary artery aneurysm (SCAA) with a *Z*‐worst ≥2.5 to <5; medium coronary artery aneurysm (MCAA): ≥5 to <10, with absolute dimension <8 mm; large or giant coronary artery aneurysm (GCAA): ≥10, or absolute dimension ≥8 mm; PLT, platelet; ESR, erythrocyte sedimentation rate; CRP: C‐reactive protein; HGB, hemoglobin; MPV, mean platelet volume; PDW, platelet distribution width; WBC, white blood cell; NA, not applicable; NS, not significant. *p < 0.05, **p < 0.01, ***p < 0.001, ****p < 0.0001 versus HS; ^####^ *p* < 0.0001 versus Acute KD.

### 2.2 Hyperactive Kawasaki Disease Platelets Are Prone to Interact with Monocytes

**Figure 2:**
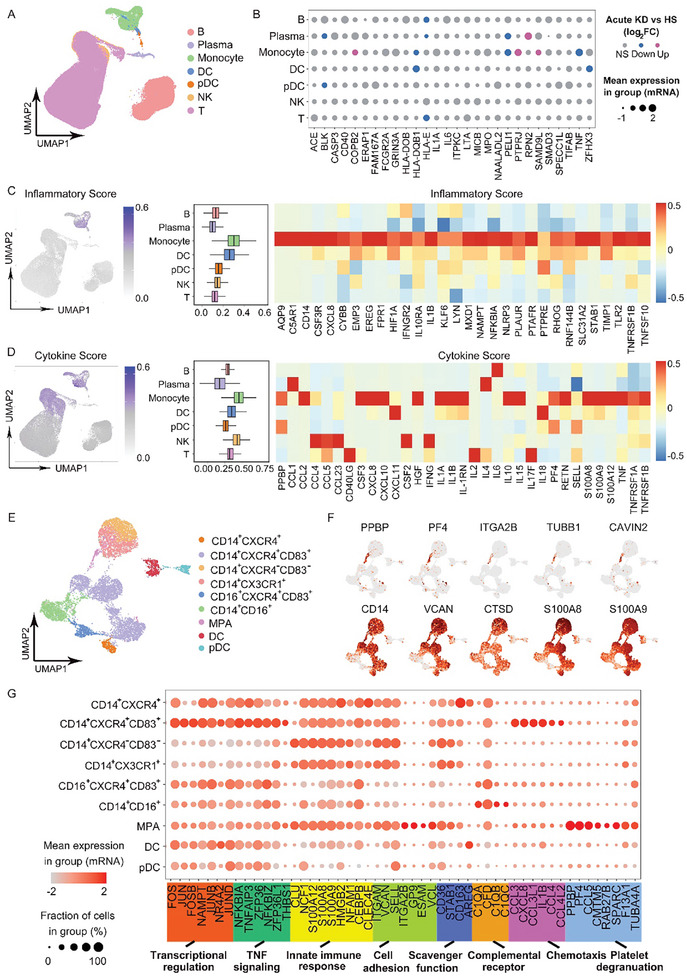
MPA contributes higher risk to cytokine storm during acute KD. A) UMAP plot visualization of peripheral blood immune cells colored by annotated cell types. Each point represents a single cell, and the cell types were annotated and colored based on 3′ gene expression. B) Dot plot showing the expression of KD‐associated risk genes in each major cell type. The color is scaled by log_2_FC of acute KD versus HS, log_2_FC > 0 label as red, log_2_FC < 0 label as blue. The dot size is proportional to the mean expression of genes associated with KD identified in recent GWAS studies. C) UMAP plot of total cells colored by inflammatory score. The gene set termed “HALLMARK_INFLAMMATORY_RESPONSE” from MsigDB. Box plots showing the inflammatory score of cell subtypes (left panel), and heatmap depicting the average normalized expression of genes significantly upregulated in monocytes (right panel). D) UMAP plot of total cells colored by cytokine score. The cytokine genes were collected based on the references of Kawasaki disease (Table S3, Supporting Information). Box plots showing the cytokine score of cell subtypes (left panel), and heatmap depicting the average normalized expression of cytokine genes reported elevation in KD in each cell types (right panel). E) UMAP visualization of monocytic lineage separated into 9 subtypes. F) UMAP visualization of MPA colored by platelet marker genes (*PPBP*, *PF4*, *ITGA2B*, *TUBB1*, *CAVIN2*), and monocyte marker genes (*CD14*, *VCAN*, *CTSD*, *S100A8*, *S100A9*). G) Dot plots of top50 marker gene expression involved in pathways including transcriptional regulation, TNF signaling, innate immune response, cell adhesion, scavenger function, complemental receptor, chemotaxis, and platelet degranulation in each monocytic subtype. HS, healthy subject; KD, Kawasaki disease; PLT, platelet; MPA, platelet–monocyte aggregate; GWAS, genome‐wide association study; FC, flod change.

We performed single‐cell RNA sequencing (scRNA‐Seq with 10× Genomics) on PBMCs from acute KD (*n* = 7), recovered KD (*n* = 6), and HS (*n* = 7) to characterize the populations of monocytes and their crosstalk with platelets in peripheral blood. Following standard data processing and quality control procedures, an average of 7524 cells per sample were sequenced (minimum of 5543 cells and <20% mitochondria reads per cell). Next, using uniform manifold approximation and projection (UMAP), seven major cell types were defined in the PBMCs, including B cells (CD79A, MS4A1 (membrane spanning 4‐domainsA1), CD19), plasma cells (JCHAIN (immunoglobulin J chain), MZB1 (margunal zone B and B1 cell specific protein 1), CD79A, CD19), monocytes (*VCAN* (versican), FCN1 (ficolin 1)), dendritic cells (DCs) (CD1C, CLEC10A (C‐type lectin domain family 10A), plasmacytoid dendritic cells (pDCs) (JCHAIN, CLEC4C, LILRA4 (leukocyte immunoglobulin like receptor A4)), nature killer (NK) cells (NCAM1 (neural cell adhesion molecule 1), CD3D, KLRC1 (killer cell lectin like receptor C1)), and T cells (CD3D, CD3E, CD3G) (**Figures** **2**A and S1A (Supporting Information)). The acute KD group exhibited an expansion of monocytes when compared with those of the HS group. However, this expansion was attenuated in recovered KD (Figure S1B, Supporting Information).

**Figure 3:**
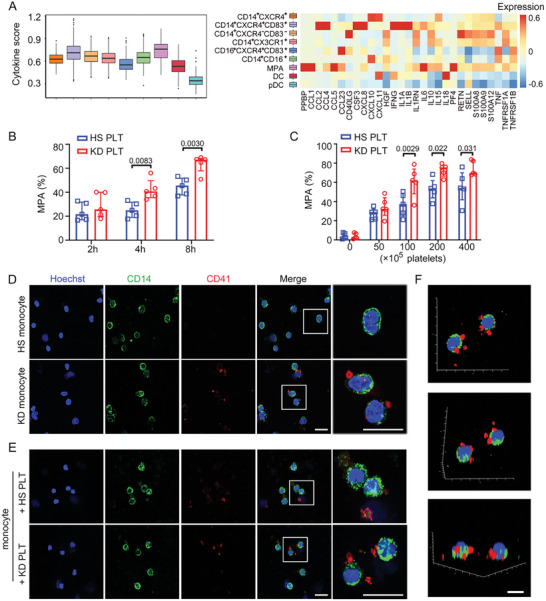
Hyperactive KD platelets are prone to crosstalk with monocytes. A) Box plot of the cytokine score in monocytes from each subtype. Significance was evaluated with the Wilcoxon rank‐sum test. B) Isolated monocytes from HS were cocultured with HS or KD platelets (1 × 10^7^) for 2, 4, and 8 h (*n* = 5). The ratio of MPA was shown. Two‐way ANOVA and Sidak's multiple comparisons test. C) Isolated monocytes from HS were cocultured with HS or KD platelets for 8 h (*n* = 5). The ratio of MPA was shown. Two‐way ANOVA and Sidak's multiple comparisons test. D,E) Representative immunofluorescence of monocytes isolated from HS and KD subjects (D), or incubated with HS and KD platelets (E), *CD14* stained as green, CD41 as red, and nuclei visualized with Hoechst (blue), *n* = 6. Scale bar: 20 µm. F) 3D reconstruction of confocal *Z*‐stack images of monocytes cocultured with KD platelets (*n* = 6). *CD14* stained as green, CD41 as red, and nuclei visualized with Hoechst (blue). Scale bar: 10 µm.

The expression of a set of candidate risk genes for KD reported previously^[^ ^18^ ^]^ was significantly changed in monocytes (Figure 2B). Notably, because hypercytokinemia is the hallmark of KD,^[^ ^19^ ^]^ we compared the inflammatory and cytokine scores (e.g., IL‐1α, IL‐1β, *S100A8 (S100 calcium binding protein A8)*, etc.), a measurement of the level of inflammatory genes and cytokines that predicts the severity of inflammation among the cell subtypes. The monocytes showed the highest inflammatory (Figure 2C) and cytokine scores (Figure 2D), suggesting that monocytes serve as the major immune effector cells in the acute phase of KD. Following reclustering of blood mononuclear phagocytes, we identified nonclassical (CD16^+^CXCR4^+^CD83^+^) (CXCR4, C‐X‐C motif chemokine receptor 4), intermediate (CD14^+^CD16^+^), and classical monocyte subtypes (including CD14^+^CXCR4^+^, CD14^+^CXCR4^+^CD83^+^, CD14^+^CXCR4^−^CD83^−^, CD14^+^CX3CR1^+^), along with an unusual subtype annotated as MPA. This subtype had the coexpression of monocyte marker genes *CD14*, *VCAN*, *CTSD (cathepsin D)*, *S100A8*, *S100A9*; and platelet marker genes *PPBP (pro‐platelet basic protein)*, *PF4(platelet factor 4)*, *ITGA2B (integrin subunit alpha 2b)*, *TUBB1 (tubulin beta‐1 chain)*, *CAVIN2 (caveolae associated protein 2)* (Figures 2E,F andS1C (Supporting Information)). Consistent with the flow cytometry analysis, the proportion of the MPA subtype was significantly increased in acute KD, and restored to HS level in recovered KD (Figure S1D, Supporting Information). The MPA subtype showed high expression of genes involved in innate immune response, cell adhesion, and platelet degranulation, confirming the characteristics of both monocytes and platelets (Figure 2G). Furthermore, MPA showed a significant increase of cytokine score compared to other subtypes during acute KD (**Figure** **3**A and Table S1 (Supporting Information)), characterized by SELL (selectin L),^[^ ^20^ ^]^ *S100A8*,^[^ ^21^ ^]^ *S100A9*,^[^ ^21^ ^]^ S100A12,^[^ ^21b^ ^]^ suggesting the proinflammatory role of MPA during acute KD.

We hypothesized that KD platelets interact with monocytes during the acute phase considering the hyperactivity of platelets during acute KD. The isolated monocytes from HS were cocultured with HS or KD platelets. Notably, the MPA ratio was significantly increased after incubation of monocytes with 1 × 10^7^ KD platelets at 4 and 8 h, respectively (Figure 3B). Furthermore, the formation of MPA increased with increasing concentration of KD platelets (8 h incubation) (Figure 3C). As shown in Figures 3D,E and S2A,B (Supporting Information), there was no direct interaction of platelets and monocytes in monocytes isolated from HS and monocytes after incubation with HS platelets. However, interactions with CD41‐labeled platelets (red) were observed in monocytes isolated from patients with KD. This interaction was more often found in monocytes incubated with KD platelets (Figures 3E andS2C,D (Supporting Information)). Additionally, we found that KD platelets were localized peripherally on monocytes after an 8 h incubation using confocal microscopy with 3D reconstruction (Figure 3F).

### 2.3 Kawasaki Disease Platelets Bind to Monocytes via the Interaction of CD62p with P‐selectin Glycoprotein Ligand 1 (PSGL‐1) and Glycoprotein (GP)Ibα with CD11b

**Figure 4:**
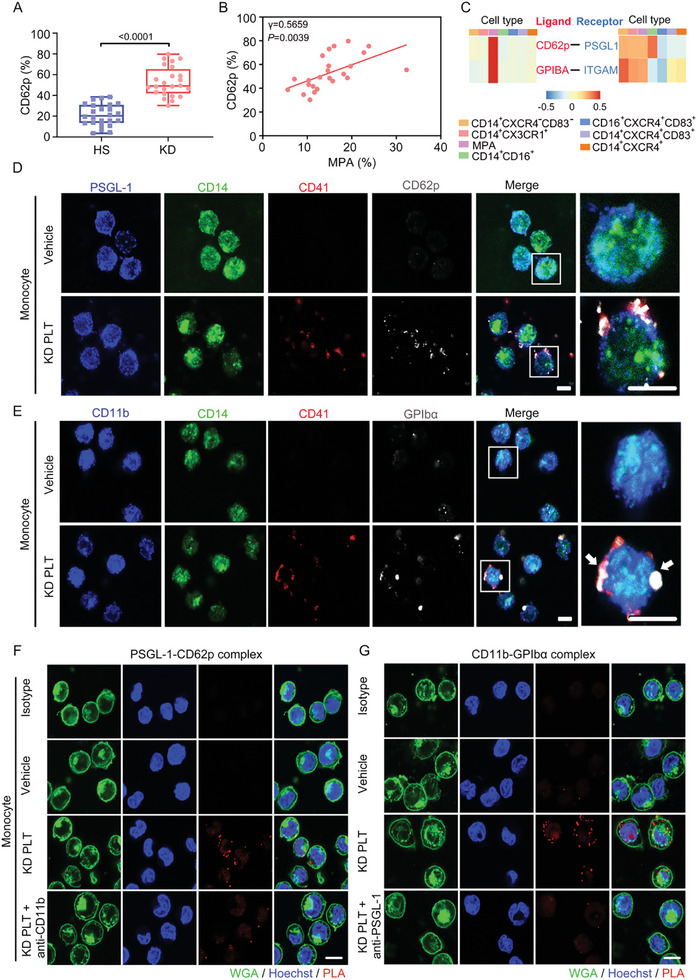
KD platelets crosstalk with monocytes via forming “adhesion junctions”. A) Flow cytometry analysis showing CD62p expression in platelets from HS (*n* = 24) and KD patients (*n* = 24). Unpaired *t* test. B) Correlation between MPA and CD62p positive platelets in patients with acute KD (*n* = 24). *p* value was calculated using correlation analysis. C) Heatmap showing selected ligand–receptor interactions between MPA and subtypes of monocytes. D) Immunostaining of PSGL‐1 (blue), *CD14* (green), CD41 (red), and CD62p (gray) in monocytes after coculture with KD platelets for 8 h. Activated CD41^+^ platelets (red) forming CD62p (gray) and PSGL‐1‐mediated junctions (blue) with CD14^+^ monocytes (green) are shown (*n* = 6). Scale bar: 5 µm. E) Immunostaining of CD11b (blue), *CD14* (green), CD41 (red), GPIbα (gray) in monocytes after coculture with KD platelets for 8 h. Activated CD41^+^ platelets (red) forming GPIbα (gray) and CD11b‐mediated junctions (blue) with CD14^+^ monocytes (green) are shown (*n* = 6). Scale bar: 5 µm. F,G) Proximity ligation assay (PLA) showing adhesion junctions of CD62p–PSGL‐1 (F), GPIbα–CD11b (G) on monocytes. PLA staining: red; nuclei: blue; wheat germ agglutinin (WGA): green (*n* = 5). Scale bar: 10 µm. PLT, platelet; HS, healthy subject; KD, Kawasaki disease; MPA, platelet–monocyte aggregate; ITGAM, integrin subunit alpha M (CD11b).

The surface expression of CD62p was significantly increased in platelets from patients with acute KD compared with those of HSs (**Figure** **4**A), and positively correlated with MPA in these patients (Figure 4B). Furthermore, the interaction of GPIbα with CD11b and CD62p with PSGL‐1 contributed to the binding of activated platelets to monocytes.^[^ ^22^ ^]^ Notably, CD62p and GPIbα were consistently derived from cluster 8 (MPA) (Figure 4C). Subsequently, they interacted with PSGL‐1 and CD11b in monocytes, respectively. Moreover, our confocal microscopy revealed that after overnight coculture, KD platelets adhered to monocytes, with platelet‐expressed CD62p colocalized at the platelet–monocyte interface, potentially binding to monocyte PSGL‐1 (Figure 4D). Furthermore, platelet‐expressed GPIbα appeared to bind to monocyte CD11b at the platelet–monocyte interfaces (Figure 4E). We performed proximity ligation assays (PLAs) to confirm the direct interaction between CD62p/PSGL‐1 and GPIbα/CD11b. PLA is a novel method for detecting direct protein–protein interactions. It operates by employing oligonucleotide‐labeled antibodies (PLA probes), which generate a signal only when the two probes are close (<30nm) (Figure S2E, Supporting Information). There was no fluorescence in the vehicle‐treated monocytes in the ligation of CD62p/PSGL‐1 and GPIbα/CD11b (Figure 4F,G). We observed an interaction between CD62p and PSGL‐1 on the monocyte surface in the presence of KD platelets; however, the interaction was attenuated by an antibody against CD11b (Figures 4F and S2F (Supporting Information)). Additionally, GPIbα on KD platelets bound to CD11b on the monocytes, and this binding was significantly inhibited by an anti‐PSGL‐1 antibody (Figures 4G and S2G (Supporting Information)), suggesting that CD62p/PSGL‐1 partnership initiates platelet–monocyte aggregation, and CD11b/GPIbα partnership further stabilizes MPA.

### 2.4 Blockade of MPA Formation Protects from the Development of Kawasaki Disease Vasculopathy

We adopted the LCWE‐induced KD murine model to mimic human KD vasculopathy.^[^ ^3^, ^23^ ^]^ LCWE‐injected mice exhibited vasculopathy of the abdominal aorta (Figure S3A,C, Supporting Information) and coronary arteries (Figure S3B,D, Supporting Information), which was characterized by immune cell infiltration, elastin degradation, and medial thickening. The levels of inflammatory cytokines, including IL‐1β (Figure S3E, Supporting Information), TNF‐α (Figure S3F, Supporting Information), IL‐6 (Figure S3G, Supporting Information), and IL‐1α (Figure S3H, Supporting Information), were significantly increased in LCWE‐induced mice 2 weeks after receiving LCWE injection, compared with the phosphate‐buffered‐saline (PBS)‐injected mice. The MPA ratio (Figure S3I, Supporting Information) and the intermediate monocytes (labeled as Ly6C^int^) (Figure S3J, Supporting Information) were significantly elevated at 2 weeks after LCWE injection.

**Figure 5:**
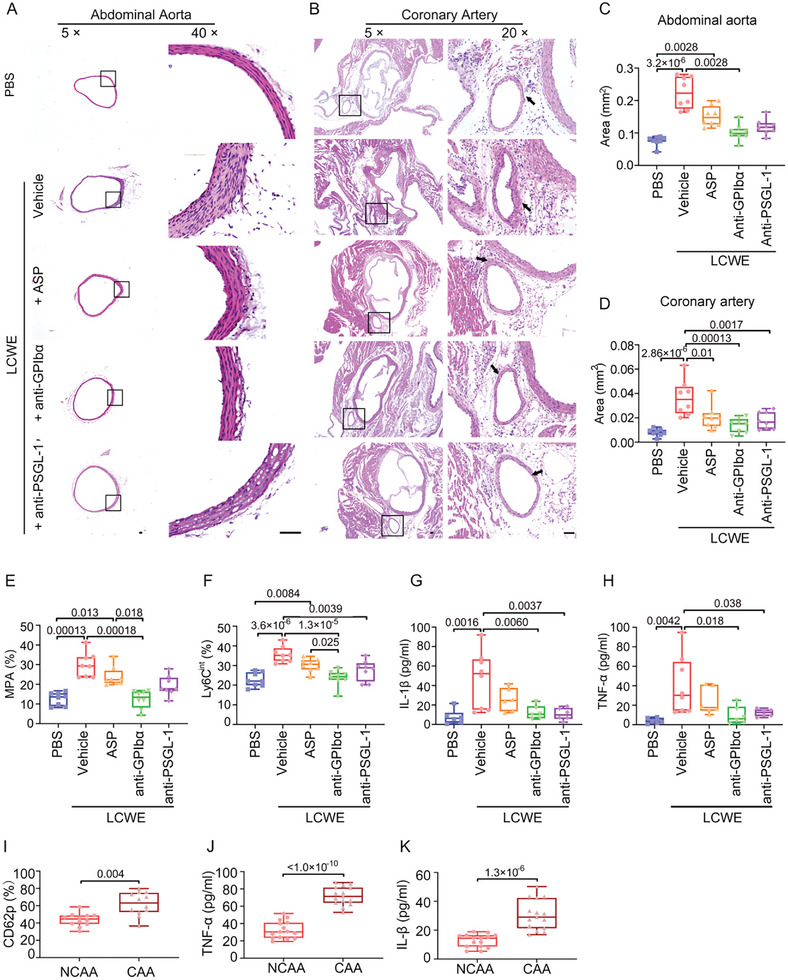
Disruption of MPA formation protects from the development of KD vasculopathy. A,B) Representative H&E staining of the abdominal aorta (A) and coronary artery (B) from PBS (*n* = 8) and LCWE‐injected mice (*n* = 8), LCWE‐injected mice followed by administration with aspirin (ASP) (*n* = 8), anti‐GPIbα (*n* = 8), anti‐PSGL‐1 (*n* = 8) were shown. Scale bar: 50 µm. C,D) Quantification of the areas of thickened media layer in the abdominal aorta (C) and coronary artery (D) from each group. Kruskal–Wallis test and Dunn's multiple comparisons test in (C), one‐way ANOVA and Tukey's multiple comparisons test in (D). E,F) Bar plots showing MPA (E) and Ly6C^int^ monocytes (F) in peripheral blood from PBS‐injected (*n* = 8) and LCWE‐injected mice (*n* = 8), LCWE‐injected mice followed by administration with ASP (*n* = 8), anti‐GPIbα (*n* = 8), anti‐PSGL‐1 (*n* = 8). Kruskal–Wallis test and Dunn's multiple comparisons test in (E), one‐way ANOVA and Tukey's multiple comparisons test in (F). G,H) Levels of IL‐1β (G), TNF‐α (H) in plasma from PBS‐injected mice (*n* = 6), LCWE‐injected mice (*n* = 8), LCWE‐injected mice followed by administration with ASP (*n* = 6), anti‐GPIbα (*n* = 6), anti‐PSGL‐1 (*n* = 6). One‐way ANOVA and Tukey's multiple comparisons test. I) Flow cytometry analysis showing the surface expression of CD62p on platelets from patients with NCAA (*n* = 12) and CAA (*n* = 12). Unpaired *t* test. J,K) Levels of TNF‐α (J), and IL‐1β (K) in plasma from patients with NCAA and CAA (*n* = 15). Unpaired *t* test. LCWE, *L. casei* cell wall extract; PLT, platelet; ASP, aspirin; MPA, platelet–monocyte aggregate; NCAA, noncoronary artery aneurysm; CAA, coronary artery aneurysm.

We administered monoclonal antibodies against PSGL‐1 and GPIbα, the day after the mice received LCWE injection to confirm our hypothesis that the crosstalk of KD platelets and monocytes contributes to LCWE‐induced KD vasculopathy. Two weeks after LCWE treatment, we found that LCWE‐induced medial thickening and damage in both abdominal aorta (**Figure** **5**A,C) and coronary artery (Figure 5B,D) were significantly attenuated by administering anti‐GPIbα. The attenuation of medial thickening was also significant in the LCWE‐injected mice treated with anti‐PSGL‐1 (Figure 5A–D). Hence, anti‐GPIbα and anti‐PSGL‐1 treatments significantly attenuated the elevation of MPA (Figure 5E) and Ly6C^int^ monocytes (Figure 5F) in the LCWE‐injected mice. Our result showed that administering anti‐GPIbα and anti‐PSGL‐1 reduced the levels of IL‐1β (Figure 5G), TNF‐α (Figure 5H), and the active and total TGFβ1 (Figure S3K,L, Supporting Information) in the LCWE‐injected mice. In line with KD murine model, during acute KD, the platelets from patients with CAA showed increased CD62p surface expression when compared with those in the NCAA group (Figure 5I). The levels of TNF‐α (Figure 5J) and IL‐1β (Figure 5K) in plasma from patients with CAA were significantly higher than those in the NCAA group.

### 2.5 Platelet–Monocyte Aggregate Formation Promotes the Conversion of Classical Monocytes (CD14^+^CD16^−^) into CD16^+^ Monocytes

**Figure 6:**
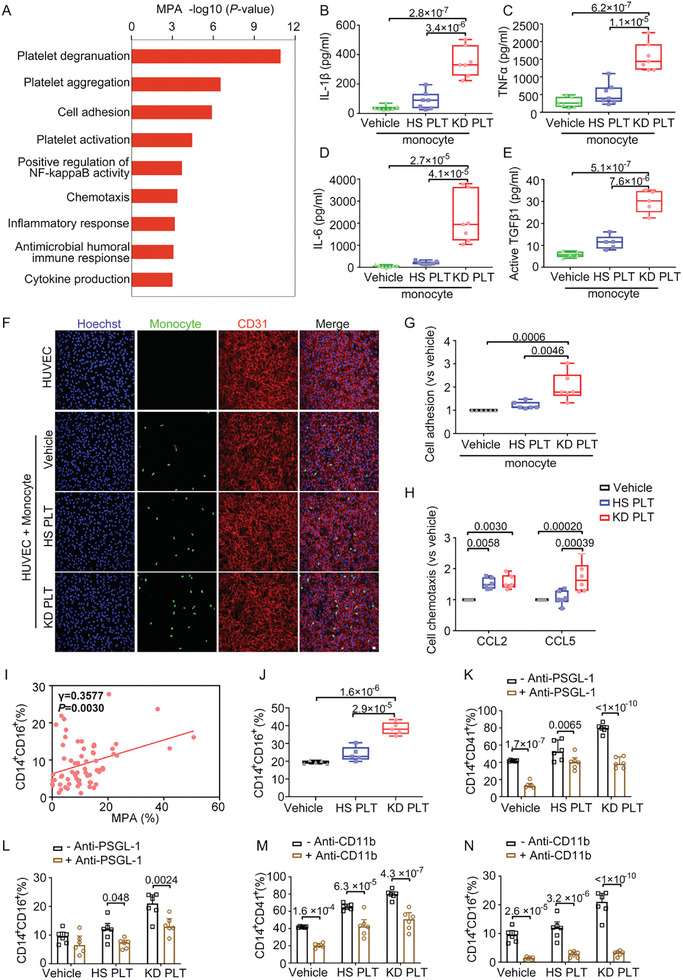
KD platelets skew circulating monocytes toward a proinflammatory phenotype via the formation of “adhesion junctions”. A) Enriched gene ontology (GO) terms in genes highly expressed in MPA. B–E) Levels of IL‐1β (B), TNF‐α (C), IL‐6 (D) (*n* = 7), and active TGFβ1 (E) (*n* = 5) in cell‐free supernatants from coculture of monocytes and HS or KD platelets were evaluated by LEGENDplex assay. One‐way ANOVA and Tukey's multiple comparisons test. F) Monocytes were incubated with HS or KD platelets for 16 h and then cocultured with TNF‐α‐treated HUVECs for 2 h, immunostaining of CMFDA‐labeled monocytes (green), CD31 (red), nuclei (blue) in HUVECs were shown (*n* = 6). Scale bar: 20 µm. G) Quantification of CMFDA‐labeled monocytes adhesion to HUVECs versus vehicle (*n* = 6). One‐way ANOVA and Tukey's multiple comparisons test. H) Monocytes were cultured with HS or KD platelets for 16 h, and the comparative value of monocytes migrated in response to CCL2 and CCL5 versus vehicle (*n* = 6). *p* values were calculated by two‐way ANOVA and Tukey's multiple comparisons test. I) Correlation between the ratio of MPA and CD14^+^CD16^+^ monocytes in PBMCs from KD patients (*n* = 67). *p* value was calculated using correlation analysis. J) Monocytes were cultured alone or with HS platelets and KD platelets for 16 h, and the CD14^+^CD16^+^ monocyte was evaluated by flow cytometry analysis (*n* = 5). K,L) Flow cytometry analysis of CD14^+^CD41^+^ (K), CD14^+^CD16^+^ monocytes (L) after coculture with HS or KD platelets in the presence of antibody against PSGL‐1 (*n* = 6). Two‐way ANOVA and Sidak's multiple comparisons test. (M,N) Flow cytometry analysis of CD14^+^CD41^+^ (M), CD14^+^CD16^+^ monocytes (N) after coculture with HS or KD platelets in the presence of antibody against CD11b (*n* = 6). Two‐way ANOVA and Sidak's multiple comparisons test. PLT, platelet; HS, healthy subject; KD, Kawasaki disease; MPA, platelet–monocyte aggregate; CCL2, C─C motif chemokine ligand 2; CCL5, C─C motif chemokine ligand 5.

The gene ontology (GO) analysis showed that MPA genes were enriched for cell adhesion, chemotaxis, and cytokine production (**Figure** **6**A). Furthermore, our result showed that the release of IL‐1β (Figure 6B), TNF‐α (Figure 6C), IL‐6 (Figure 6D), and active TGFβ1 (Figure 6E) was significantly increased in monocytes incubated with KD platelets compared with those with HS platelets.

TNF‐α is known to induce an inflammatory response in vascular endothelial cells by promoting leukocyte adhesion in KD vasculitis.^[^ ^24^ ^]^ Notably, monocytes incubated with KD platelets showed greater adhesion to TNF‐α‐pretreated endothelial cells (Figure 6F,G). Additionally, these monocytes exhibited a marked increase in migratory ability in response to C─C motif chemokine ligand 5 (CCL5) when compared with those incubated with HS platelets (Figure 6H).

We applied partition‐based graph abstraction (PAGA) to visualize the developmental trajectory of cell subcluster within a graph framework to explore the transitional relationships across monocyte subtypes. CD14^+^CXCR4^−^CD83^−^ monocytes were differentiated into two distinct fates, fate 1: MPA followed by CD14^+^CD16^+^, and fate 2: CD14^+^CX3CR1^+^ followed by CD14^+^CD83^+^CXCR4^+^ subtypes (Figure S4A, Supporting Information). We constructed a pseudotime trajectory map to further validate the dynamic transition process of monocytes from CD14^+^CXCR4^−^CD83^−^ into MPA as the intermediate state and into CD14^+^CD16^+^ as a terminally differentiated state during acute KD (Figure S4B, Supporting Information). Notably, single‐cell transcriptomic analysis along the trajectory revealed that genes in group 2 were upregulated in the MPA state and contributed to the positive regulation of monocyte differentiation and leukocyte cell–cell adhesion (Figure S4C, Supporting Information). Overall, these results suggest MPA subtype acts as the critical transitional state for exacerbating inflammation in fate 1.

The proportion of MPA positively correlated with the levels of CD14^+^CD16^+^ in patients with acute KD (Figure 6I). Additionally, incubation with KD platelets increased both MPA levels and the number of CD14^+^CD16^+^ monocytes (Figure 6J), which was confirmed using confocal microscopy (Figure S4D, Supporting Information). Reports revealed that activated platelets converted CD14^+^CD16^−^ into CD14^+^CD16^+^ monocytes.^[^ ^25^ ^]^ Our results also showed that the addition of antibodies to the coculture system against PSGL‐1 or CD11b almost completely prevented MPA formation (Figure 6K,M) and subsequent CD16 expression (Figure 6L,N). We further confirmed the role of adhesion synapse formed by CD62p and PSGL‐1 using CD62p‐deficient mice. The results showed complete silencing of CD62p expression in platelets (Figure S5A,B, Supporting Information). Furthermore, CD62p‐deficient mice exhibited a significant reduction of MPA ratio (Figure S5C, Supporting Information) and Ly6C^int^ monocytes (Figure S5D, Supporting Information). In the LCWE‐induced murine model, medial thickening was alleviated in both abdominal aortas (Figure S5E,G, Supporting Information) and coronary arteries (Figure S5F,H, Supporting Information) of CD62p deficient mice, confirming the key role of platelet CD62p in KD vasculopathy.

### 2.6 Platelet–Monocyte Aggregate Formation Induces Active TGFβ1 Release and Nuclear Factor kappaB (NFκB) Nuclear Translocation in Monocytes

**Figure 7:**
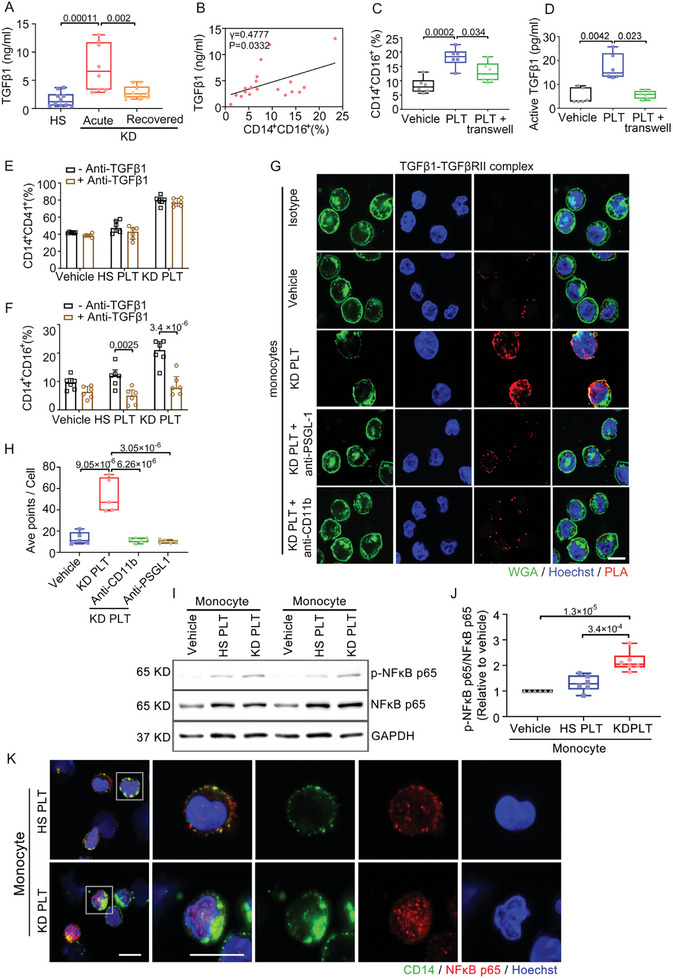
“Adhesion junctions” between KD platelets and monocytes induced TGFβ release and NFκB nuclear translocation in monocytes. A) Box plot showing levels of TGFβ1 in plasma from participants. HS: *n* = 10, Acute KD: *n* = 8, Recovered KD: *n* = 10. B) Correlation between CD14^+^CD16^+^ monocytes and plasma TGFβ1 in KD patients (*n* = 18). *p* value was calculated using correlation analysis. C) Flow cytometry analysis of the CD14^+^CD16^+^ monocytes after direct coculture with KD platelets, or in a transwell system for 16 h (*n* = 6). One‐way ANOVA and Tukey's multiple comparisons test. D) The level of active TGFβ1 in the supernatant of monocytes after direct coculture with KD platelets, or in a transwell system for 16 h (*n* = 6). One‐way ANOVA and Tukey's multiple comparisons test. E,F) Flow cytometry analysis of CD14^+^CD41^+^ (E), CD14^+^CD16^+^ monocytes (F) after coculture with HS or KD platelets in the presence of antibody against TGFβ1 (*n* = 6). Two‐way ANOVA and Sidak's multiple comparisons test. G) PLA showing adhesion junctions of TGFβ1–TGFβRII on monocytes. PLA staining: red; nuclei: blue; WGA: green. Scale bar: 10 µm. H) Quantification of average points per cell in each group determined by ImageJ, and subjected to statistical analysis for significance. One‐way ANOVA and Tukey's multiple comparisons test. I,J) The expression (I) and quantification (J) of phosphorylated NFκB and total NFκB in monocytes cocultured with HS or KD platelets were determined by Western blot (*n* = 6). *p* values were calculated by one‐way ANOVA and Tukey's multiple comparisons test. K) Representative immunofluorescence images of nuclear translocation of NFκB in monocytes isolated from HS and KD patients (*n* = 5). *CD14* was stained as green, NFκB as red, and nuclei were visualized with Hoechst (blue). Scale bar: 10 µm. HS, healthy subject; KD, patients with Kawasaki disease; PLT, platelets.

MPA marker genes were highly enriched in inflammation‐related signaling pathways, such as a positive regulation of NFkappaB transcription factor activity. Upon analyzing the intersection of genes, we identified TGFβ1, *S100A8*, *S100A9*, and S100A12 in the two pathways (Figure S6A, Supporting Information). Notably, the addition of TGFβ1 significantly induced CD16 expression on CD14^+^ monocytes (Figure S6B, Supporting Information). Moreover, we found a significant increase of TGFβ in plasma from acute KD (Acute KD vs HS, *p* = 0.00011), which reduced to a lower level comparable to HS in the plasma of patients from recovered KD (Acute KD vs Recovered KD, *p* = 0.002) (**Figure** **7**A). The level of TGFβ was positively correlated with the ratio of CD14^+^CD16^+^ monocytes (*p* = 0.0332) (Figure 7B), suggesting the role of TGFβ in phenotypic alteration of monocytes. Furthermore, we found that CD14^+^CD16^+^ monocytes were significantly reduced when platelets and monocytes were physically separated by a transwell system (Figure 7C). An increased level of active TGFβ1 was found after coculture with KD platelets; however, such increase of active TGFβ1 was significantly reduced by the transwell system (Figure 7D), suggesting that direct interaction of KD platelet and monocyte induces more production of active TGFβ1. Administering TGFβ1 antibody in the coculture system decreased only CD16 expression and not MPA formation (Figure 7E,F). PLA analysis further showed that interaction between TGFβ1 and TGFβRII was significantly increased after incubation with KD platelets. Meanwhile, blocking either PSGL‐1 or CD11b significantly reduced the interaction of TGFβ1 and TGFβRII, suggesting that direct cell–cell interaction induces more TGFβ1 release (Figure 7G,H). We also showed that monocytes incubated with platelets had increased NFκB phosphorylation (Figure 7I,J), and monocytes incubated with KD platelets exhibited an increase in NFκB nuclear translocation as revealed by confocal imaging (Figure 7K). This finding was consistent with our GO pathway analysis (Figure 6A).

### 2.7 Silencing of Platelet TGFβ1 Protects from Platelet–Monocyte Aggregate‐Mediated Kawasaki Disease Vasculopathy

**Figure 8:**
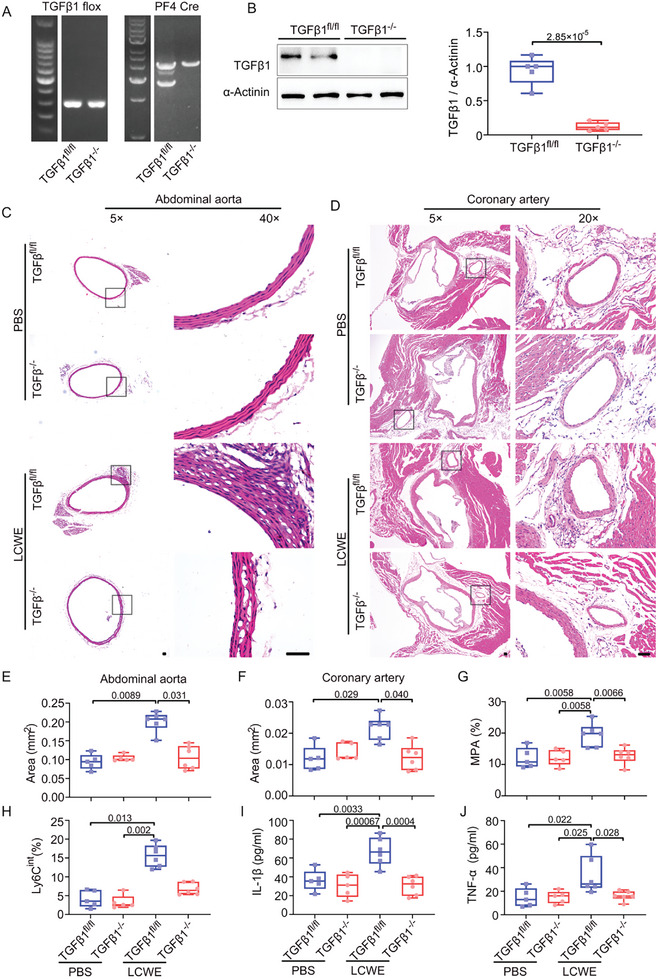
Silencing of platelet TGFβ1 protects from MPA‐mediated KD vasculopathy. A) Verification of TGFβ1 excision in hematopoietic cells using genomic PCR. B) The expression and quantification of TGFβ1 in platelets isolated from Plt–TGFβ1 KO (*PF4*‐Cre: TGFβ1^fl/fl^) mice and TGFβ1 flox (TGFβ1^fl/fl^) mice were determined by Western blot (*n* = 5). Unpaired *t* test. C,D) Representative hematoxylin and eosin (H&E) staining of the abdominal aorta (C) and coronary artery (D) from TGFβ1 flox and Plt–TGFβ1 KO mice injected with PBS (*n* = 5) or LCWE (*n* = 6) were shown. Scale bar: 50 µm. E,F) Quantification of the areas of thickened media layer in the abdominal aorta (E) and coronary artery (F) from each group. Kruskal–Wallis test and Dunn's multiple comparisons test. G,H) Bar plots showing MPA (G) and Ly6C^int^ monocytes (H) in peripheral blood from PBS‐injected TGFβ1 flox (*n* = 5), PBS‐injected Plt–TGFβ1 KO mice (*n* = 5), LCWE‐injected TGFβ1 flox (*n* = 6), and LCWE‐injected Plt–TGFβ1 KO mice (*n* = 6). One‐way ANOVA and Tukey's multiple comparisons test in (G), Kruskal–Wallis test and Dunn's multiple comparisons test in (H). I,J) Levels of IL‐1β (I), TNF‐α (J) in plasma from PBS‐injected TGFβ1^fl/fl^ (*n* = 5), PBS‐injected Plt–TGFβ1 KO mice (*n* = 5), LCWE‐injected TGFβ1 flox (*n* = 6), and LCWE‐injected Plt–TGFβ1 KO mice (*n* = 6). One‐way ANOVA and Tukey's multiple comparisons test. LCWE, *L. casei* cell wall extract; PLT, platelet; ASP, aspirin; MPA, platelet–monocyte aggregate; Plt–TGFβ1 KO, *PF4*‐Cre: TGFβ1^fl/fl^; TGFβ1 flox, TGFβ1^fl/fl^.

Platelet (Plt)–TGFβ1 knock out (KO) (TGFβ1^fl/fl^ *PF4*‐Cre) mice were generated and administered with LCWE to determine whether platelet TGFβ was required for MPA‐mediated vasculopathy in vivo. **Figure** **8**A shows the efficient targeting of the TGFβ1 locus in megakaryocytes and platelets. Moreover, the protein expression of TGFβ1 was almost undetectable in platelets from Plt–TGFβ1 KO mice compared with those in TGFβ flox mice (TGFβ1^fl/fl^) (Figure 8B), confirming a complete silencing of TGFβ1 expression in platelets. No significant difference was found in platelet count (Figure S7A, Supporting Information), platelet activity (Figure S7B, Supporting Information), and tail bleeding time (Figure S7C, Supporting Information) of Plt–TGFβ1 KO mice compared with that of TGFβ flox. The levels of total and active TGFβ1 increased, two weeks after LCWE injection, corroborating the results from patients with KD (Figure S3K,L, Supporting Information). Compared with the PBS‐treated mice, LCWE injection in TGFβ1 flox mice significantly enhanced the infiltration of immune cells and medial thickening in the abdominal aorta (Figure 8C,E) and coronary artery (Figure 8D,F); however, these effects were not found in LCWE‐injected Plt–TGFβ1 KO mice. A consistently significant elevation of MPA ratio and Ly6C^int^ monocytes was detected in TGFβ1 flox mice two weeks after LCWE injection; however, the changes were attenuated in Plt–TGFβ1 KO mice (Figure 8G,H). Moreover, Plt–TGFβ1 KO mice showed significantly decreased levels of IL‐1β (Figure 8I) and TNF‐α (Figure 8J) in plasma after LCWE injection compared with those in control. Furthermore, our in vitro results showed that suppression of TGFβ1 resulted in the reduction of CD14^+^CD16^+^ monocytes; however, it did not affect MPA formation (Figure 7E,F). Conversely, MPA was significantly reduced in Plt–TGFβ1 KO mice (Figure 8G,H). This discrepancy suggests that platelet‐derived TGFβ1 may contribute to platelet activation and resultant MPA formation in vivo. Overall, our results reveal that platelet TGFβ1 provides a positive feedback loop between platelets and monocytes, which amplifies the inflammation, consequently leading to KD‐induced vasculopathy (Central Illustration: the adhesion‐mediated platelet–monocyte positive feedback loop exacerbates inflammation and vasculopathy of KD. TNF‐α stimulates KD platelets ①, leading to elevated mitochondrial ROS (mtROS) and reduced mitochondrial membrane potential (∆Ψm) ②, which results in platelet activation ③and increased surface expression of CD62p, this in turn interacts with PSGL‐1 on monocytes ④. The adhesion synapse was further stabilized by the binding of platelet GPIbα to monocyte CD11b ⑤, resulting in the release of platelet TGFβ1⑥. The platelet‐derived TGFβ1 promotes the phenotypic conversion of CD14^+^ monocytes into a proinflammatory phenotype ⑨ (including the release of IL‐1β and TNF‐α) by increasing NFκB phosphorylation ⑦ and nuclear translocation ⑧. This in turn activates the platelets ⑩, forming a positive feedback loop between platelets and monocytes.).

## 3 Discussion

Platelet–monocyte interaction, in general, is vital for monocyte function, which has been reported in patients with various diseases. In addition, this interaction is positively correlated with the severity of the disease.^[^ ^26^ ^]^ The pathological features and clinical complications of KD are hyperactive platelets and systemic inflammation.^[^ ^4^ ^]^ Recently, one study revealed that platelet activation exacerbates vascular inflammation via MPA formation, leading to KD vasculitis in LCWE‐induced murine models.^[^ ^16^ ^]^ However, the mechanism by which MPA contributes to the etiology and pathogenesis of cardiovascular disease, especially in KD, remains unclear. Herein, we report that the level of MPA was significantly increased in acute KD, especially in patients with CAA. Notably, hyperactive KD platelets skewed the CD14^+^ monocytes toward the proinflammatory CD14^+^CD16^+^ phenotype through an initial interaction with CD62p/PSGL‐1, followed by stabilization through a junction with GPIbα/CD11b. In a positive feedback mechanism, activated monocytes, in turn, induce platelet activation leading to TGFβ1 release and monocyte nuclear localization of nuclear factor B. Furthermore, the complete silencing of platelet TGFβ1 (breaking the positive feedback loop) significantly promoted the resolution of inflammation and vasculopathy in the LCWE‐induced murine model. Overall, our results suggest that the formation of MPA contributes to the hyperinflammatory state in KD. Thus, targeting MPA or reducing platelet TGFβ1 might provide novel therapeutic approaches for KD vasculopathy.

MPA formation may contribute to the pathogenesis of various vascular diseases by promoting inflammation and thrombosis. It was found to be significantly elevated in patients with acute coronary syndromes. Additionally, it promotes the adhesion of monocytes to the endothelium, thereby contributing to inflammation and thrombosis.^[^ ^27^ ^]^ Furthermore, another study found that MPA was significantly elevated in patients with unstable angina, myocardial infarction,^[^ ^28^ ^]^ and acute ischemic stroke.^[^ ^29^ ^]^ Recent studies show that platelets skew monocytes toward proinflammatory phenotype in septic mice^[^ ^30^ ^]^ and that the genetic depletion of platelets or treatment with an anti‐GPIbα antibody significantly reduced LCWE‐induced cardiovascular lesions by blocking MPA formation.^[^ ^16^ ^]^ We also found that monocytes incubated with KD platelets exhibited a marked increase in migratory ability in response to CCL5. Since CD16^+^ monocytes express heightened levels of CCR5 (C‐C chemokine receptor type 5),^[^ ^7^ ^]^ which is the ligand for CCL5, these findings suggest that MPA may contribute to the infiltration of proinflammatory macrophages in the vascular wall. In a recent study, platelets were found to induce both inflammasome activation and IL‐1 production of monocytes.^[^ ^31^ ^]^ A previous study also showed that global caspase‐1 knockout mice exhibited less LCWE‐induced coronary lesions.^[^ ^23c^ ^]^ In our KD patients, neither platelets nor monocytes exhibited activation of caspase‐1 and NLRP3 inflammasome compared to HSs (data not shown).

CD62p and GPIbα are the platelet activation markers and important modulators of platelet–monocyte interaction.^[^ ^32^ ^]^ Moreover, previous studies have reported that GPIbα becomes internalized during platelet activation and observed a decrease in the labeling on the platelet surface.^[^ ^33^ ^]^ However, other studies identified a significant increase in GPIbα density after thrombin treatment observed as labeling on fully spread and surface‐activated platelets.^[^ ^34^ ^]^ White et al. later reported that dilation of the open canalicular system in thrombin‐activated platelets would facilitate the expression of GPIbα sites recognized by antibodies, hence, leading to a more intense labeling of GPIbα on the surface of activated platelets.^[^ ^35^ ^]^ In our study, we highlighted that platelet CD62p and GPIbα formed adhesion synapses by binding to monocyte PSGL‐1 and CD11b. These interactive platelet‐mediated “bridges” between platelets and monocytes resemble the immunological synapse between antigen‐presenting cells (APCs) and lymphocytes, which activates monocytes and resultant contact‐dependent monocytic polarization. The antibody targeting αM (P201‐K217) reduces αMβ2‐dependent adhesion to GPIbα, but not to other ligands, suggesting the important role of CD11b in Mac‐1 (also known as integrin αMβ2, or CD11b/CD18) during interaction with platelet GPIbα.^[^ ^36^ ^]^ In vitro, the binding of activated platelets to leukocytes significantly increased the surface expression of Mac‐1.^[^ ^37^ ^]^ Furthermore, sequestration of surface‐exposed PSGL‐1 decreases platelet–leukocyte interaction,^[^ ^38^ ^]^ suggesting that the binding of P‐selectin and PSGL‐1 was the initial step, which increased the expression of Mac‐1 on monocytes, leading to the binding of GPIbα and Mac‐1.^[^ ^22a^ ^]^ In our study, both CD62p/PSGL‐1 and CD11b/GPIbα partnership were found in MPA formation; however, disrupting each partnership using antibodies inhibited the formation of MPA. Therefore, our results suggest that the CD62p/PSGL‐1 partnership initiates platelet–monocyte aggregation, whereas the CD11b/GPIbα partnership stabilizes MPA. Notably, in our LCWE‐induced KD murine model, administering aspirin decreased the ratio of MPA and Ly6C^int^ monocytes, which led to reduced KD vasculopathy. As aspirin irreversibly inhibits co‐oxygenase enzymes and resultant platelet activation,^[^ ^39^ ^]^ its therapeutic effect in KD was at least in part resulting from the inhibition of MPA formation. Notably, Inclacumab, a fully human anti‐P‐selectin antibody, has shown safety in sustained anticell adhesion effects in atherosclerotic cardiovascular diseases.^[^ ^40^ ^]^ Our results in the KD murine model support the potential of Inclacumab in treating KD vascular lesions. Overall, we reveal that the adhesion synapse between platelets and monocytes serves as a dynamic adaptive controller that regulates the functional responses of monocytes and that targeting the adhesion‐mediated platelet–monocyte positive feedback loop alleviates KD‐induced inflammation and vasculopathy.

Additionally, TGFβ has been previously reported in thickened intima from coronary artery tissues of KD autopsies; however, it was not detected in age‐matched control coronary arteries,^[^ ^41^ ^]^ suggesting that TGFβ may contribute to the pathogenesis of KD. Furthermore, platelets store high levels of TGFβ in the alpha granule in an inactive high molecular weight complex, which becomes activated after it is released into the extracellular environment.^[^ ^42^ ^]^ In addition, platelets are known to constitutively express the cell‐surface‐docking receptor, glycoprotein A repetitions predominant (GARP) for TGFβ1, and upregulate its expression upon activation.^[^ ^43^ ^]^ Studies have supported that platelet GARP plays a dominant role in activating TGFβ1 in circulation,^[^ ^43^, ^44^ ^]^ consistent with the previous study showing that platelet‐derived TGFβ induced CD16 expression in monocytes.^[^ ^45^ ^]^ Our results further identify TGFβ1 as the key initiator of monocyte phenotypic alteration, beginning with the interaction of P‐selectin/PSGL‐1 and followed by stabilization of GPIbα/CD11b. In the LCWE‐induced murine model, inhibition of MPA formation by administering anti‐GPIbα or anti‐PSGL‐1 significantly reduced the plasma level of active TGFβ1. The deficiency of platelet TGFβ significantly decreased the ratio of CD14^+^CD16^+^ monocytes and systemic inflammation. Furthermore, the presence of the TGFβ‐anchoring GARP protein on platelets is a unique feature except for Treg cells. Hence, platelets may contribute to inflammation via the formation of adhesion synapses with monocytes to locally activate latent TGFβ and resultant monocytes. A prior study with the LCWE‐induced KD murine model reported that inhibiting TGFβ does not affect inflammatory cell infiltration;^[^ ^46^ ^]^ however, this discrepancy can be explained because in the previous study, neonatal mice (7 days) were used, and TGFβ was blocked with an antibody, whereas we used 4 weeks old platelet‐specific TGFβ knockout mice in our study.

Consistent with our findings, the levels of *S100A8*/*A9* and S100A12, released by activated neutrophils and monocytes, were previously reported to be significantly elevated in patients with KD, particularly during the acute phase of the disease.^[^ ^47^ ^]^ Furthermore, the levels of S100 proteins in patients with KD were found to correlate with disease severity and the risk of developing coronary artery lesions in KD.^[^ ^21b^ ^]^ Furthermore, the elevated levels of S100 proteins in these patients may suggest that these proteins contribute to the inflammation and vascular damage associated with KD by promoting the production of proinflammatory cytokines, which enhances the adhesion and migration of immune cells to the sites of inflammation. Notably, a previous study reported that TGFβ induces the expression of *S100A8*/*A9* in human monocytes,^[^ ^48^ ^]^ which provides a possible link between TGFβ and *S100A8*/*A9*, indicating that platelet‐derived TGFβ may activate the proinflammatory monocytes by inducing expression of *S100A8*/*A9* in KD. Our scRNA‐Seq libraries generated by 10× Genomics did not include neutrophils due to low RNA content; therefore, further studies that will examine the role of platelet–neutrophil aggregates in KD‐induced inflammation and vasculopathy are needed.

The reason why KD platelets are hyperactive remains unclear, yet one recent study identified TNF‐α as the key proinflammatory cytokine promoting platelet hyperactivity via elevation of mitochondrial mass and oxygen consumption.^[^ ^49^ ^]^ Additionally, TNF‐α is an inflammatory cytokine that plays an important role in KD vasculitis. Several studies have established that blocking TNF‐α has significantly improved the treatment of children with KD.^[^ ^50^ ^]^ Pignatelli et al. reported that TNF‐α behaved as an aggregating agent by eliciting platelet aggregation and TxB2 formation in collagen‐primed platelets.^[^ ^51^ ^]^ We found that the plasma levels of TNF‐α in patients with KD and LCWE‐induced KD mice were higher than those in healthy subjects and control mice (Figure S8A,B, Supporting Information), and KD platelets exhibited elevated mitochondrial ROS (Figure S8C, Supporting Information) and reduced membrane potential (Figure S8D, Supporting Information), respectively. In addition, the plasma level of TGFβ was higher in patients with KD (Figure S8E, Supporting Information), especially those with CAA (Figure S8F, Supporting Information). Therefore, a high level of TNF‐α in KD may contribute to platelet hyperactivation and resultant MPA formation. We administered monoclonal antibody infliximab against TNF‐α in our KD murine model to confirm TNF‐α contribution to MPA and systemic inflammation. The results showed that infliximab attenuated the vascular pathology in abdominal aortas (Figure S8G,I, Supporting Information) and coronary arteries (Figure S8H,J, Supporting Information) with concomitant reduction of MPA (Figure S8K, Supporting Information) and Ly6Cint monocytes (Figure S8L, Supporting Information) in the LCWE‐injected mice, suggesting that TNF‐α contributes to MPA and the resultant vascular pathology during acute KD.

Overall, our study findings reveal that activated platelets interact with monocytes through the formation of an adhesion synapse driven by the engagement of platelet CD62p/GPIbα with monocyte PSGL‐1/CD11b, which promotes TGFβ1 release and activation, hence, directing the phenotypic alteration of monocytes into CD14^+^CD16^+^ monocytes. The CD14^+^CD16^+^ monocytes, in turn, release inflammatory cytokines, thereby activating platelets and eventually forming an adhesion‐mediated platelet–monocyte positive feedback loop. Our study represents a valuable resource for understanding peripheral immunity in KD and provides new evidence for the critical immunomodulatory role of hyperactive platelets in KD vasculopathy. However, larger clinical studies are needed to establish the use of MPA as a biomarker for CAA.

## 4 Experimental Section

### Animal Experiments

All the animal procedures were performed by the same operator who was blinded to the mouse genotype/treatment group for the in vivo animal model experiments. The experiments were conducted under the authorization of the Animal Care Committee at the Guangzhou Medical University (2019‐384), and the Guangdong Medical Laboratory Animal Center (C202306‐38).

### Ethics Statement

Ethical approval was obtained from the Medical Ethics Committee of the Guangzhou Women and Children's Medical Center ([2022]203A01). The implementations followed the International Ethical Guidelines for Research Involving Human Subjects as stated in the Helsinki Declaration ([2023]CJ0050). Informed consent was obtained from the guardians of all participants.

### Participants

Patients diagnosed with KD (American Heart Association criteria)^[^ ^52^ ^]^ were randomly recruited from the Guangzhou Women and Children's Medical Center. Participants with underlying congenital heart disease, such as bicuspid aortic valve, mitral valve prolapses, and/or hemodynamically insignificant ventricular septal defects, were excluded. All recruited patients with KD were treated with high‐dose IVIG (2 g kg^−1^) as a single infusion within 10 days of illness onset; however, after diagnosis, low‐dose aspirin (3 to 5 mg kg^−1^ per day) was continued as soon as possible until no evidence of coronary abnormalities after onset of illness. For patients with acute KD, blood samples (3mL) were collected for MPA test during their initial visit to the hospital following the onset of fever, before the IVIG treatment. Patients were further evaluated for the presence of CAA based on the echocardiogram; for the recovered patients with KD, blood samples were taken at least 21 days after aspirin and IVIG therapy. All the blood samples were freshly processed for experimental use, and coronary artery aneurysm was determined using the score of coronary angiography, expressed as *Z*‐worst, which was the inner diameter of the proximal coronary artery segment/body surface area. A normal coronary artery dimension was defined as a *Z*‐worst <2.5. For small coronary artery aneurysm (SCAA), a *Z*‐worst ≥2.5 to <5 was used; medium coronary artery aneurysm (MCAA) was determined as ≥5 to <10, with absolute dimension <8 mm; and large or giant coronary artery aneurysm (GCAA) was defined as ≥10, or absolute dimension ≥8 mm. Age‐ and gender‐matched healthy children were used as HSs. The demographic and clinical characteristics of the recruited participants are presented in Table 1. The number of biological samples used in flow cytometry analysis (*n* = 20 for HS; *n* = 14 for acute KD; *n* = 20 for recovered KD), single cell sequencing (*n* = 7 for HS; *n* = 7 for acute KD; *n* = 6 for recovered KD), in vitro experiments (*n* = 40 for HS; *n* = 35 for acute KD), and diagnostic cohort (*n* = 60 for HS; *n* = 32 for acute KD; *n* = 60 for recovered KD). The “*n*” number listed in each experiment indicated the number of biological samples from participants.

### Mice

The CD62p global knockout mice (CD62p KO), platelet‐specific TGFβ1 KO mice (TGFβ1^flox/flox^ *PF4*‐Cre) generated by crossing TGFβ1^flox/flox^ and *PF4*‐Cre mice were purchased from Shanghai Model Organisms Center, Inc., China. Next, genotyping was performed via polymerase chain reaction (PCR) using standard methods; the primer sequences are listed in Table S2 (Supporting Information). Mice were fully backcrossed at least 10 generations on the C57BL/6 background. The male mice between 4–5 weeks of age were randomly selected for experiments.

### Cell Culture

Human acute monocytic leukemia cell line (THP‐1) obtained from Fu Heng Biology Inc. China were cultured in the roswell park memorial institute (RPMI)1640 medium supplemented with 10% fetal bovine serum (FBS, GIBCO, USA). Furthermore, human umbilical vein endothelial cells (HUVECs) (PriMed‐iCELL‐002) purchased from iCell Bioscience Inc., China, were used between passages 3 and 6 and grown in iCell Primary Endothelial Cell Culture System, supplemented with 10% FBS and 1% penicillin/streptomycin at 37 °C in 5% carbon dioxide at 100% humidity.

### Peripheral Blood Mononuclear Cells

Human PBMCs were isolated from the sodium citrate solution anticoagulated venous blood of participants using Histopaque‐1077 (Sigma‐Aldrich) following standard density gradient centrifugation methods. All blood samples were processed within 2 h of sample collection. The PBMCs, serum, and monocytes were separated, collected, or frozen for later processing. Murine peripheral blood (≈0.6 mL) was collected from the right cardiac ventricle into containers containing 100 µL of 3.2% sodium citrate (Leagene, China). The murine PBMCs were obtained using density gradient centrifugation (Solarbio, China) based on the manufacturer's instructions. Subsequently, red blood cell lysis was performed (TIANGEN, China).

### CD14^+^ Monocytes

CD14^+^ monocytes were isolated from PBMCs using anti‐CD14 conjugated microbeads through magnetic‐automated cell sorting (Miltenyi Biotec). The purity of isolated monocytes was assessed on a Sysmex XN‐350 hematology analyzer and confirmed to be more >90.0%. The collected monocytes were seeded on the surfaces of poly‐l‐lysine‐coated 14 mm coverslips or directly in 24‐well plates with RPMI 1640 supplemented with 10% FBS and 1% penicillin/streptomycin at a concentration of 2 × 10^5^ mL^−1^. After incubating at 1:100 with HS/KD platelets for 8 or 16 h, the resulting cell suspension was transferred to a clean polypropylene tube, and the cells were collected by centrifuging at 100 *g* for 5 min for flow cytometry analysis; Alternatively, the 24‐well plates were centrifuged at 100 *g* for 5 min, and cells were harvested on coverslips, washed, and fixed with 4% paraformaldehyde for immunofluorescence staining.

### Single‐Cell Suspension Preparation

PBMCs were suspended in 1× PBS containing 0.02% bovine serum albumin. The 0.4% trypan blue was used to assess cell viability under microscopic observation. Cells with >80% viability qualified for the library construction process.

### Library Construction and Single‐Cell RNA Sequencing

The scRNA‐Seq libraries were generated using the 10× Genomics Chromium Controller Instrument and Chromium Single Cell 3′ V2 Reagent Kits (10× Genomics, Pleasanton, CA, USA). Briefly, the prepared single‐cell suspension was loaded into each channel to generate single‐cell Gel Bead‐In‐Emulsions (GEMs). After the reverse transcription (RT) step, GEMs were broken, and barcoded complementary DNA (cDNA) was purified and amplified. The amplified barcoded cDNA was fragmented, A‐tailed at the 3′ end, ligated with adaptors, and amplified using PCR. The final libraries were quantified using the Qubit High Sensitivity DNA assay (Thermo Fisher Scientific), and the size distribution for each library was determined using a High Sensitivity DNA chip on an Agilent 2100 bioanalyzer (Agilent Technologies, Santa Clara, CA, USA). All libraries were sequenced as 100 bp paired‐end reads on Illumina sequencer (MGISEQ‐2000, BGI, China).

### Alignment and Quality Control of Sequencing Data

Raw data were processed using fastq with default parameters to filter adaptor sequences and remove the low‐quality reads. The unique‐molecular‐identifier (UMI)‐based clean data were mapped to the human reference genome (GRCh38 Ensemble: version 100) using the STAR algorithm with customized parameters from the UMI‐tools standard pipeline. Finally, the combined raw expression matrix generated from each sample was aggregated using CellRanger (v3.1.0). Cells containing over 200 expressed genes and mitochondria UMI rate below 20% passed the cell quality filtering, with mitochondria genes subsequently removed from the expression table. Potential doublets were identified and removed using Doublet Detection.

### Identification of Major Cell Clusters

The Seurat package (version: 3.1.4, https://satijalab.org/seurat/) was used for cell normalization, data scaling, clustering, and most of the visualization. Additionally, principal component analysis was constructed based on the scaled data with the 2000 highly variable genes to reduce the dimensionality of the expression matrix. The first 20 principal components were used for UMAP construction to embed the dataset into 2D. Furthermore, the clusters were annotated to known biological cell types based on the expression of canonical markers, except for MPA, the cluster both expressed markers for monocytes (*CD14*, *VCAN*, *CTSD*, *S100A8*, and *S100A9*), and markers for platelets (*PPBP*, *PF4*, *ITGA2B*, *TUBB1*, and *CAVIN2*). Therefore, it was labeled as MPA. For each cluster, the marker genes were identified using the FindAllMarkers function with the Wilcox rank sum test algorithm under the following criteria: 1. lnFC > 0.25; 2. *p* adjust < 0.05; 3. min.pct > 0.1.

### Partition‐Based Graph Abstraction Analysis

PAGA was performed using the “PAGA connectivity measure.” This measure was a test statistic quantifying the degree of connectivity of two partitions and had a close association with modularity.^[^ ^53^ ^]^ For each pair of clusters, PAGA connectivity was the ratio of the number of interedges between the clusters normalized with the number of interedges expected under random assignment of edges. The PAGA tool in scanpy (tl.paga) was used to generate the abstracted graph between clusters.

### Pseudotime Analysis

Pseudotime transitional trajectory of monocytes in fate 1 was constructed utilizing the Monocle package (version: 2.22.0, http://cole‐trapnell‐lab.github.io/monocle‐release) with default parameters.^[^ ^54^ ^]^ The top 2000 highly variable genes in monocytes were selected as input. Furthermore, DDRTree was applied for data dimension reduction. The differentially expressed genes across subclusters were identified by the “differentialGeneTest” function with a *q*‐value <10^−20^, and visualized in 2D space using the “plot_pseudotime_heatmap” function. Pathway enrichment analysis was performed for the differentially expressed genes with Database for Annotation, Visualization, and Integrated Discovery.

### Inflammatory, Cytokine Module Score Analysis

A gene set termed “HALLMARK_INFLAMMATORY_RESPONSE” was downloaded from MsigDB^[^ ^55^ ^]^ to define the inflammatory score,  the cytokine score and cytokine genes were collected based on the references of Kawasaki disease (Table S3, Supporting Information). The Gene Set Variation Analysis (GSVA) software package^[^ ^56^ ^]^ was used to calculate the score of the predefined gene set in the respective cell. Furthermore, the R package was used to draw a box plot diagram and calculate the difference of this gene set score between one group and the mean of all the other groups based on the Wilcox rank‐sum algorithm.

### Platelet Purification

Human platelets were isolated from the venous blood of participants (healthy and participants with KD) and prepared as previously described.^[^ ^3^ ^]^ Briefly, the platelet‐rich plasma (PRP) was obtained by centrifugation at 250*g* for 20 min. After treatment with prostaglandin E1 (Sigma, 100 nm), the PRP was further centrifuged at 250*g* for 3 min to remove as many residual white blood cells as possible. The supernatants obtained were centrifuged at 1000*g* for 5 min, and the sediment of platelets was resuspended to 10^8^ platelets mL^−1^ in Hanks' Balanced Salt Solution buffer. The purity of platelet preparation was determined by flow cytometry (BD LSRFortessa X‐20) analysis using platelet markers (>99% CD41 positive).

### Flow Cytometry Analysis

Human monocytes in PBMC were identified by staining with Allophycocyanin (APC) anti‐human Lineage Cocktail (CD3, CD19, CD20, CD56) (BioLegend, 363601), fluorescein isothiocyanate anti‐human CD11b (Biolegend, 301330). The subtypes of monocytes were classified using Phycoerythrin(PE)/Cyanine7 anti‐human *CD14* (BioLegend, 367112) and PE anti‐human CD16 (BioLegend, 360704). Additionally, MPA was identified as Lin^−^ (CD3, CD19, CD20, CD56), CD11b^+^, CD14^+^, CD41^+^ (BioLegend, 303706) cells. In some experiments, human monocytes were directly incubated at 1:100 with freshly isolated platelets from HS/participants with KD. Alternatively, transwell chambers with a pore size of 0.4 µm were used to separate platelets from direct interaction with monocytes. The monocytes were collected and resuspended in fluorescence‐activated cell sorting buffer after 16 h of incubation. The expressions of *CD14* and CD16/CD41 were analyzed by BD LSRFortessa X‐20 using the FlowJo‐V10 software. For murine monocytes, PBMCs were identified by staining with Brilliant Violet 421 anti‐mouse Lineage Cocktail, including anti‐mCD3 (Biolegend, 100227), anti‐mCD45R(B220) (Biolegend, 103239), anti‐mNK1.1 (Biolegend, 108731), and anti‐mTER‐119/Erythroid cells (Biolegend, 116233). Additional markers used were PE anti‐mouse Ly6G (BioLegend, 127608), PE/Cyanine7 anti‐mouse/human CD11b (BioLegend, 101216), Alexa Fluor 488 anti‐mouse CD115 (BioLegend, 135512), APC anti‐mouse Ly6C (BioLegend, 128016). Monocytes in murine PBMC were identified as Lin^−^ (mCD3, mCD45R, mNK1.1, mTER‐119/Erythroid cells), and Ly6G^−^, CD11b^+^, subdivided into Ly6C^high^, Ly6C^int^, and Ly6C^low^ subsets. MPA was identified as Lin^−^ (mCD3, mCD45R, mNK1.1, mTER‐119/Erythroid cells), Ly6G^−^, CD11b^+^, Ly6C^+^, and CD41^+^ cells. A minimum of 1 × 10^4^ events were collected per sample. Data were analyzed with FlowJo‐V10 software (BD Biosciences).

### Proximity Ligation Assay

PLA assays were performed using the Duolink In Situ Red Starter Kit Mouse/Rabbit (Sigma‐Aldrich, DUO92102) to detect the direct interaction between CD62p and PSGL‐1, GPIbα and CD11b, TGFβ1 and TGFβRII. The assay was performed following the manufacturer's protocol. Briefly, cells were washed with PBS, fixed with 4% paraformaldehyde, and labeled with 5 µg mL^−1^ Alexa‐Fluor‐488‐conjugated wheat germ agglutinin (WGA) (ThermoFisher, Cat. W11261) for 10 min at room temperature. After blocking in Duolink blocking buffer (Sigma‐Aldrich), the cells were incubated with the primary antibody mouse anti‐CD62p (1:100, R&D systems, BBA30) and Rabbit anti‐PSGL‐1 (1:100, Abcam, ab227836) or mouse anti‐GPIbα (1:100, Novus biologicals, NBP1‐42151) and Rabbit anti‐CD11b (1:100, Abcam, ab184308), or TGFβ1 (1:100, Proteintech, 21898‐1‐AP) and TGFβRII (1:100, Abcam, ab78419). All the incubations were overnight. Subsequently, the addition of the appropriate Duolink secondary antibodies (Sigma‐Aldrich), ligation, and amplification steps of the PLA were performed following the manufacturer's instructions. The cell nuclei were visualized with Hoechst staining. The images were acquired on immunofluorescence confocal microscopy (×63 oil immersion lens, Leica SP8). Researchers were blinded to the groups, and images were assigned a numerical code to ensure that the selection of representative images was carried out with blinding. The average number of PLA spots per cell from each group was calculated by ImageJ software and subjected to statistical analysis for significance.

### Cytokine Analysis by Multiplex Bead‐Based Immunoassay

Cytokines in the cell supernatant (Human Inflammation Panel: LEGENDplex, Cat. 740809; Human Free Active/Total TGFβ1 Assay: LEGENDplex, Cat. 740450) and murine plasma (mouse Inflammation Panel: LEGENDplex, Cat. 740446; Mouse/Rat Free Active/Total TGFβ1 Assay: LEGENDplex, Cat. 740490) were measured by BioLegend's LEGENDplex bead‐based immunoassays using a flow cytometer. Human monocytes were seeded in 24‐well plates and coincubated with HS/KD platelets directly or separated in a transwell chamber. After 16 h coculture, the supernatants were collected by centrifugation at 250 × *g* for 5 min and transferred to a clean polypropylene tube. Another centrifugation at 1000 × *g* for 15 min was performed to completely remove platelets and precipitates. The TGFβ1 assay was performed following the manufacturer's instructions. Briefly, 50 µL of each sample was added to the plate and incubated at room temperature for 2 h while shaking at 200 rpm. After washing the plate a minimum of 4 times, 100 µL of TGFβ1 Detection Antibody solution was added to each well and incubated at room temperature for 1 h while shaking. After washing, 100 µL Avidin‐Horseradish Peroxidase (HRP) D solution was added and incubated for 30 min. Finally, 100 µL of Substrate Solution F was added to each well and incubated for 10 min. The reaction was stopped by adding 100 µL Stop Solution. Paired software was used for data analysis.

### Monocyte Adhesion to Endothelial Cells

Monocyte adhesion assay was performed as previously described.^[^ ^26^, ^57^ ^]^ Briefly, HUVECs were seeded at a density of 1 × 10^5^ on poly‐l‐lysine‐coated confocal dishes. THP‐1 labeled with Cell Tracker Green 5‐chloromethylfluorescein diacetate (CMFDA) (Life Technology, 1µm, 30 min, 37 °C) were cocultured with HS/KD platelets for 16 h. After treatment with TNF‐α (R&D systems, 2 ng mL^−1^) for 4 h, HUVECs were cocultured with platelet‐pretreated THP‐1 for 2 h. After washing off nonadherent monocytes with PBS, the cells were fixed with 4% paraformaldehyde solution and incubated with anti‐CD31 (1:100, Abcam, ab9498) and subsequently, the Alexa‐Fluor‐594‐conjugated Immunoglobulin G (IgG) antibody (1:200, Abcam, ab150116). Hoechst staining was performed to visualize the nuclei, and images were taken with immunofluorescence confocal microscopy (Leica SP8). The number of adhering monocytes was calculated using the ImageJ software. Each treatment with 3 complicates was calculated, and the average number of adhering monocytes per sample was used as one individual data for comparison and subjected to statistical analysis for significance.

### Monocyte Chemotaxis

Migration of monocytes toward CCL5 and CCL2 was analyzed using a transwell chamber (Corning) with a 4 µm pore polycarbonate membrane separating the upper from the lower compartment, as previously described.^[^ ^26b^ ^]^ Briefly, monocytes (1 × 10^5^ cells per well) labeled with Cell Tracker Green 5‐chloromethylfluorescein diacetate (Life Technology, 1 µm, 30 min, 37 °C) were loaded onto the upper chamber, and recombinant proteins CCL5 (MCE, HY‐P7283) and CCL2 (Peprotech, 300‐04) served as chemoattractants at a concentration of 50 ng mL^−1^, respectively, in the lower chamber. After incubation for 4 h, a multifunctional microplate detector at the excited wavelength of 492 nm and the emitted wavelength of 517 nm was used to count the fluorescence intensity of migrated cells for each well. Researchers were blinded to the status or groups, and images were assigned a numerical code to ensure that the selection of representative images was carried out with blinding. Each treatment with 3 complicates was calculated, and the average fluorescence intensity per group/treatment was used as one individual data for comparison and subjected to statistical analysis for significance.

### Kawasaki Disease Murine Model

*L. casei* (American Type Culture Collection, ATCC 11578) cell wall extract was prepared as previously described.^[^ ^3^, ^23^, ^58^ ^]^ Four or five weeks old C57BL/6 mice were intraperitoneally (i.p.) injected with 400 µg LCWE. For the treatment groups, mice were also injected with the following blocking antibodies: anti‐PSGL‐1 (Clone 4RA10; BioXCell; 200 µg per mouse by intravenous (iv) injection) once a week;^[^ ^59^ ^]^ anti‐GPIbα (R300, emfret, 50 µg per mouse by iv injection) for twice a week;^[^ ^60^ ^]^ and anti‐TGFβ1 (Clone 1D11.16.8; BioXCell; 250 µg per mouse by i.p. injection) for 3 times a week.^[^ ^61^ ^]^ Aspirin (A2093, Sigma‐Aldrich) was administered at 30 µg g^−1^ per day per mouse using a gavage.^[^ ^62^ ^]^ Two weeks after the LCWE injection, mice were sacrificed and perfused with PBS, followed by 4% paraformaldehyde. The heart and abdominal aorta were removed and embedded in an optimal cutting temperature (OCT) compound for histological analysis.

### Cytokine Analysis by Enzyme‐Linked Immunosorbent Assay (ELISA)

The levels of TNF‐α and IL‐1β in plasma from the human samples, as shown in Figure 1D,E, were determined using the Luminex xMAP system in clinical testing, whereas in Figure 5J,K, they were determined using human TNF‐α ELISA Kit (CUSABIO, CSB‐E04740h), and human IL‐1β ELISA Kit (CUSABIO, CSB‐E08053h). Briefly, 100 µL of human/mouse plasma was incubated with the capture and secondary antibodies following the manufacturer's instructions. The color intensity was measured at 450 nm.

### Western Blot Analysis

Cell lysates were prepared using protein lysis buffer containing protease inhibitor (Protease Inhibitor Cocktail Set I, Sigma‐Aldrich) and phosphatase inhibitor (PhosSTOP EASYpack, Roche). Furthermore, protein extracts of cells were separated using 10% sodium dodecyl sulfate polyacrylamide gel electrophoresis gels and transferred to polyvinylidene fluoride membranes. The membrane was then incubated with primary antibodies for p65‐NFκB (Abcam, catalog ab76302, 1:1000), NFκB (Abcam, catalog ab32536, 1:1000), and TGFβ1 (Proteintech, catalog 21898‐1‐AP, 1:1000) at 4 °C overnight. The next day, the membrane was incubated with HRP‐conjugated secondary antibodies at room temperature for 1 h. Glyceraldehyde 3‐phosphate dehysrogenase (GAPDH) (Abcam, catalog ab181602, 1:2000) and α‐actinin (Abcam, catalog ab18061, 1:3000) were used as an internal control for normalization and the protein bands were visualized using Immobilon chemiluminescent substrate (Millipore) and quantified using the Image Lab software (USA).

### Immunohistochemical Staining

The heart and abdominal aorta from mice were fixed in 4% paraformaldehyde for 20 min and transferred to 30% sucrose in PBS before embedding in the OCT compound and frozen. The 5 µm frozen sections were stained with hematoxylin–eosin (H&E). Subsequently, two slides, each with 3–6 sections were assessed for each mouse during the immunohistochemical staining. Briefly, the sections were immersed in xylene and alcohol, followed by staining with hematoxylin for 5 min and with eosin for 2 min. All the images were obtained using the same light source and were assigned a numerical code to ensure that the selection of representative images was performed with blinding. The areas circumscribed by the internal elastic lamina (IEL) and external elastic lamina (EEL) were measured by tracing along the respective vessel regions using ImageJ software. The areas of the thickened medial layer that were calculated by the difference between EEL and IEL were compared between groups and subjected to statistical analysis for significance.

### Immunofluorescence

Cells were stained with mouse anti‐human *CD14* (Abcam, ab181470), rabbit anti‐human CD41 (Abcam, ab134131), and rabbit anti‐human CD16 (Abcam, ab246222) antibodies to detect platelet–monocyte aggregates and the altered expression of CD16. Hoechst staining was performed to visualize the nuclei. Coverslips were fixed on a glass slide, and images were taken with immunofluorescence confocal microscopy (×63 oil immersion lens, Leica SP8).

Furthermore, to detect the interaction between P‐selectin and PSGL‐1, GPIbα and CD11b, monocytes were stained with anti‐human *CD14* (M5E2, BioLegend 301842), CD41 (HIP8, BioLegend 303706), CD62P (RD, BBA30), PSGL‐1 (Abcam, ab227836), CD11b (Abcam, ab184308), and CD42b/GPIbα (Novus, NBP1‐42151). Hoechst staining was performed to visualize the nuclei, and images were taken with immunofluorescence confocal microscopy (×63 oil immersion lens, Leica SP8).

Two slides, each with 3–6 sections were assessed for each group or treatment for immunofluorescent staining. Researchers were blinded to the status or groups, and images were assigned a numerical code to ensure that the selection of representative images was performed in a blinded manner. The mean fluorescent intensity was calculated using the ImageJ software and subjected to statistical analysis for significance.

### Data and Code Availability

The raw data were available from the Genome Sequence Archive for humans with accession ID: HRA004974. The materials that support the findings of this study were available from the corresponding author upon reasonable request.

### Statistical Analysis

The experimental data were obtained from at least four independent experiments and presented as medians ± IQR. Statistical analysis was performed using GraphPad Prism v.9.0 (GraphPad, La Jolla, CA, USA). Furthermore, normality was estimated using the Shapiro–Wilk normality test. For normally distributed data, the unpaired two‐tailed Student's *t* test or one‐way analysis of variance followed by using Tukey's multiple comparisons test was performed to determine differences between the groups. For data that were not distributed normally, the Mann–Whitney nonparametric test or Kruskal–Wallis followed by Dunn's multiple comparisons was performed to analyze differences between indicated groups. A receiver operating characteristic curve analysis was performed to determine the optimal cutoff and area under the curve. NS was considered nonsignificant (*p* > 0.05), * indicated *p* < 0.05, ** indicated *p* < 0.01, and *** denoted *p* < 0.001.

## Conflict of Interest

The authors declare no conflict of interest.

## Author Contributions

Y.Z., C.J., and M.G. contributed equally to this work. Y.Z. and W.H.T. conceptualized the project and designed the experiments; J.H. provided technical support in the experimental design; C.J., M.G., Q.C., T.W., and Y.X. performed the experiments; Y.W., X.F., and J.G. assisted with, or helped in the experimental data analysis and interpretation; Z.J., M.W., J.Z., D.C., L.F., and X.G. provided clinical samples, and the clinical data of enrolled subjects; Y.Z. wrote the first draft; T.Y., R.L., R.E., J.H., and W.H.T. reviewed and edited the paper. All authors provided critical comments on the paper.

## Supporting information



Supporting Information

## Data Availability

The data that support the findings of this study are openly available in [the Genome Sequence Archive for humans] at https://ngdc.cncb.ac.cn/gsa/.
