# Supplementary material for: Platelet–Monocyte Aggregate Instigates Inflammation and Vasculopathy in Kawasaki Disease
Source: Adv Sci (Weinh). 2024 Dec 12;12(5):2406282. doi: 10.1002/advs.202406282 (PMC11792051; doi:10.1002/advs.202406282)
Supplement: Supplementary file 1 — Supporting Information [file ADVS-12-2406282-s001.docx]

**Table S1. Pairwise comparison of mean cytokine score was conducted between MPA and other monocyte subtypes.**

| case | control | case_mean | control_mean | *P* value |
| --- | --- | --- | --- | --- |
| MPA | CD14_high_CXCR4+_CD83+_Mono | 0.718108605 | 0.679321896 | 4.34E-06 |
| MPA | CD14_high_Mono | 0.718108605 | 0.635386441 | 2.75E-24 |
| MPA | CD16_low_CD14_high_Mono | 0.718108605 | 0.597699115 | 2.69E-45 |
| MPA | CD16_high_Mono | 0.718108605 | 0.556011052 | 6.24E-63 |
| MPA | CD14_high_CXCR4_high | 0.718108605 | 0.553410958 | 5.75E-49 |
| MPA | DC | 0.718108605 | 0.490123722 | 7.52E-74 |
| MPA | CD16+_CXCR4+_CD83+_Mono | 0.718108605 | 0.47878664 | 1.40E-80 |
| MPA | pDC | 0.718108605 | 0.384724314 | 4.97E-73 |

**Table S1. Pairwise comparison of mean cytokine score was conducted between MPA and other monocyte subtypes.** Footnotes: The Wilcox rank-sum algorithm was used for statistical analysis, and *P*<0.05 was defined as statistical significance. Abbreviations: MPA, platelet-monocyte aggregate.

**Table S2. Primers for RT-qPCR.**

| **Primer** | **Forward Sequence** | **Reverse Sequence** |
| --- | --- | --- |
| SELP-P1+P2 | TGAGTTGTGCACGTCTTGGA | GGCGTGTCTGCCTTTCCTAA |
| SELP-P3+P4 | CTACAGTGTGCTCTGTGGCA | GCAGCCAGCCTGAAACAAAA |
| TGFβ1 Flox | AAGAGCAGTCAAGATAACCAGCTG | CATCCGTGTGTCTGTGTGCAGAGT |
| PF4 Cre | GCCCAGTAGTAGAGCACTTCCT | CCATAGCTGTTCTGGCACTAACTC |

**Table S3. Collected cytokine genes based on the references of kawasaki disease.**

| **References** | **Cytokine genes** |
| --- | --- |
| Multisystem inflammatory syndrome in children and Kawasaki disease: a critical comparison. (Nat Rev Rheumatol, 2021) | IL-1β, TNF, IL-8 |
| An Artificial Intelligence-guided signature reveals the shared host immune response in MIS-C and Kawasaki disease.(Nat Commun, 2022) | IL-1β, TNF-α, IL-8, IL-10 |
| CXCL10/IP-10 Is a Biomarker and Mediator for Kawasaki Disease.(Circ Res, 2015) | IL-17F, CD40L, E-selectin, CCL23, CXCL10 |
| Similarities and differences between the immunopathogenesis of COVID-19–related pediatric multisystem inflammatory syndrome and Kawasaki disease.(J Clin Invest, 2021) | IFN-γ, IL-18, IP-10, MCP-1, IL-1α, IL-1RA, RANTES, GM-CSF, IL-10 |
| Raised serum interleukin 15 levels in Kawasaki disease.(Ann Rheum Dis, 2003) | IL-15 |
| Monocyte-Derived Interleukin-1β As the Driver of S100A12-Induced Sterile Inflammatory Activation of Human Coronary Artery Endothelial Cells: Implications for the Pathogenesis of Kawasaki Disease.(Arthritis Rheumatol, 2019) | IL-1β, S100A12 |
| Cytokine Cascade in Kawasaki Disease Versus Kawasaki-Like Syndrome.(Physiol Res,2022) | IL-1β, IL-2, IL-4, IL-6, IL-10, IFN-γ, TNF-α |
| Unique activation status of peripheral blood mononuclear cells at acute phase of Kawasaki disease.(Clin Exp Immunol, 2010) | IL-10, TNF, IFN-γ, CCL2, CCL4, CCL5, CSF3, VEGFA, HGF, S100A9, S100A12 |
| Expression of myeloid-related protein-8 and -14 in patients with acute Kawasaki disease (J Am Coll Cardiol, 2006). | S100A9, S100A8 |
| Monocyte-Derived Interleukin-1β As the Driver of S100A12-Induced Sterile Inflammatory Activation of Human Coronary Artery Endothelial Cells: Implications for the Pathogenesis of Kawasaki Disease. (Arthritis Rheumatol, 2019) | S100A12, IL-1β |
| Serum resistin concentrations in children with Kawasaki disease. (Inflamm Res, 2010) | RETN |
| Reduced Platelet miR-223 Induction in Kawasaki Disease Leads to Severe Coronary Artery Pathology Through a miR-223/PDGFRbeta Vascular Smooth Muscle Cell Axis.(Circ Res, 2020) | PF4, PPBP |
| Pediatric Kawasaki Disease and Adult Human Immunodeficiency Virus Kawasaki-Like Syndrome Are Likely the Same Malady.(Open Forum Infect Dis, 2016) | TNFRSF1A, TNFRSF1B, CCL1, CCL2, CXCL11 |
| Expression of Eosinophilic Subtype Markers in Patients with Kawasaki Disease. (Int J Mol Sci, 2022) | SELL |

**Figure S1**

**
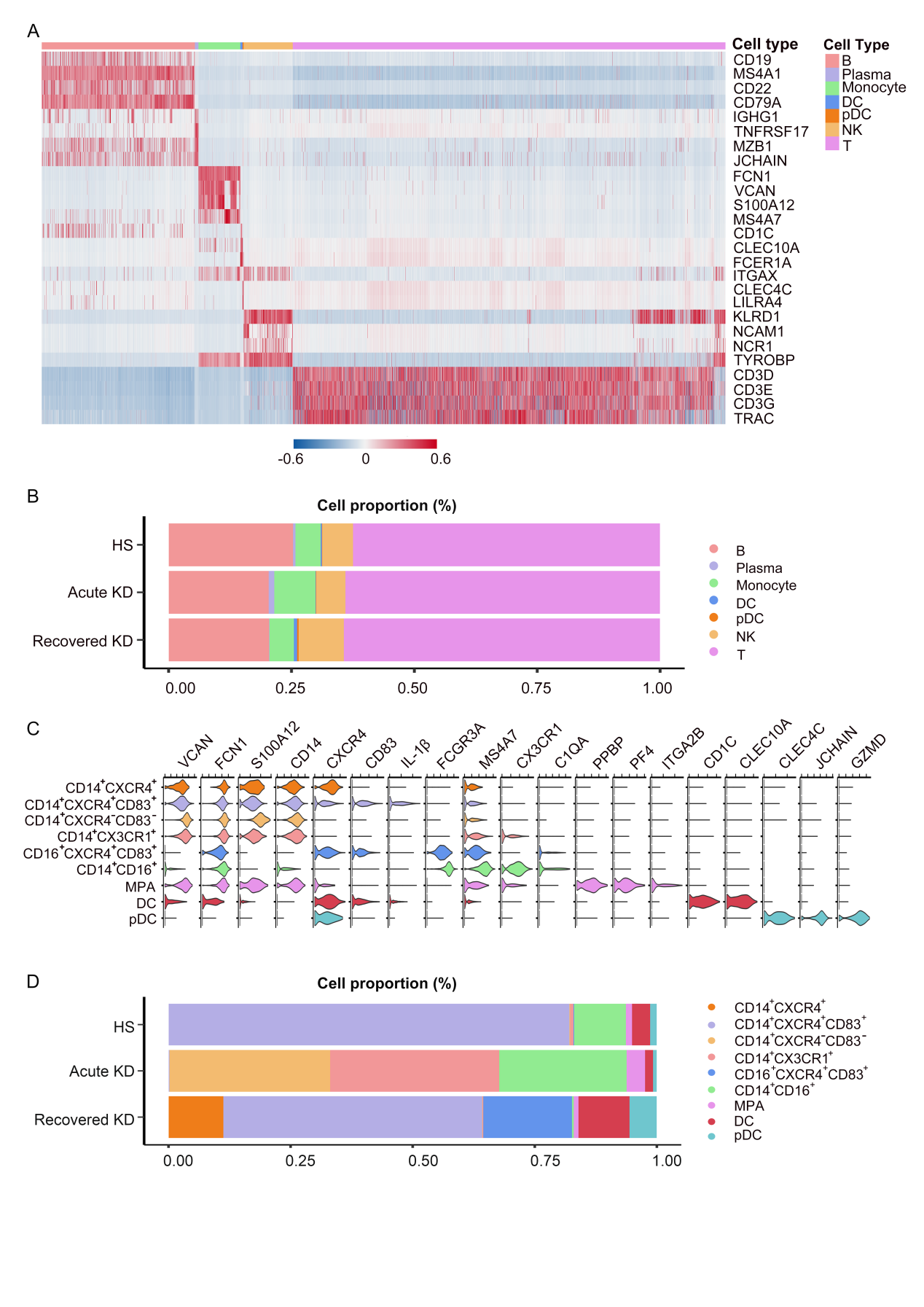
**

**Figure S1.** **Single-cell transcriptome profiling of PBMCs from participants.**

1. Heatmap displaying cell type-specific marker genes.
2. Bar plots showing the proportion of cell types separated by groups.
3. Violin plots showing marker genes for each subtype in monocytes.
4. Bar plots showing the proportion of each subtype separated by groups.

HS, healthy subject; KD, patients with Kawasaki disease; MPA, platelet-monocyte aggregate.

**Figure S2**


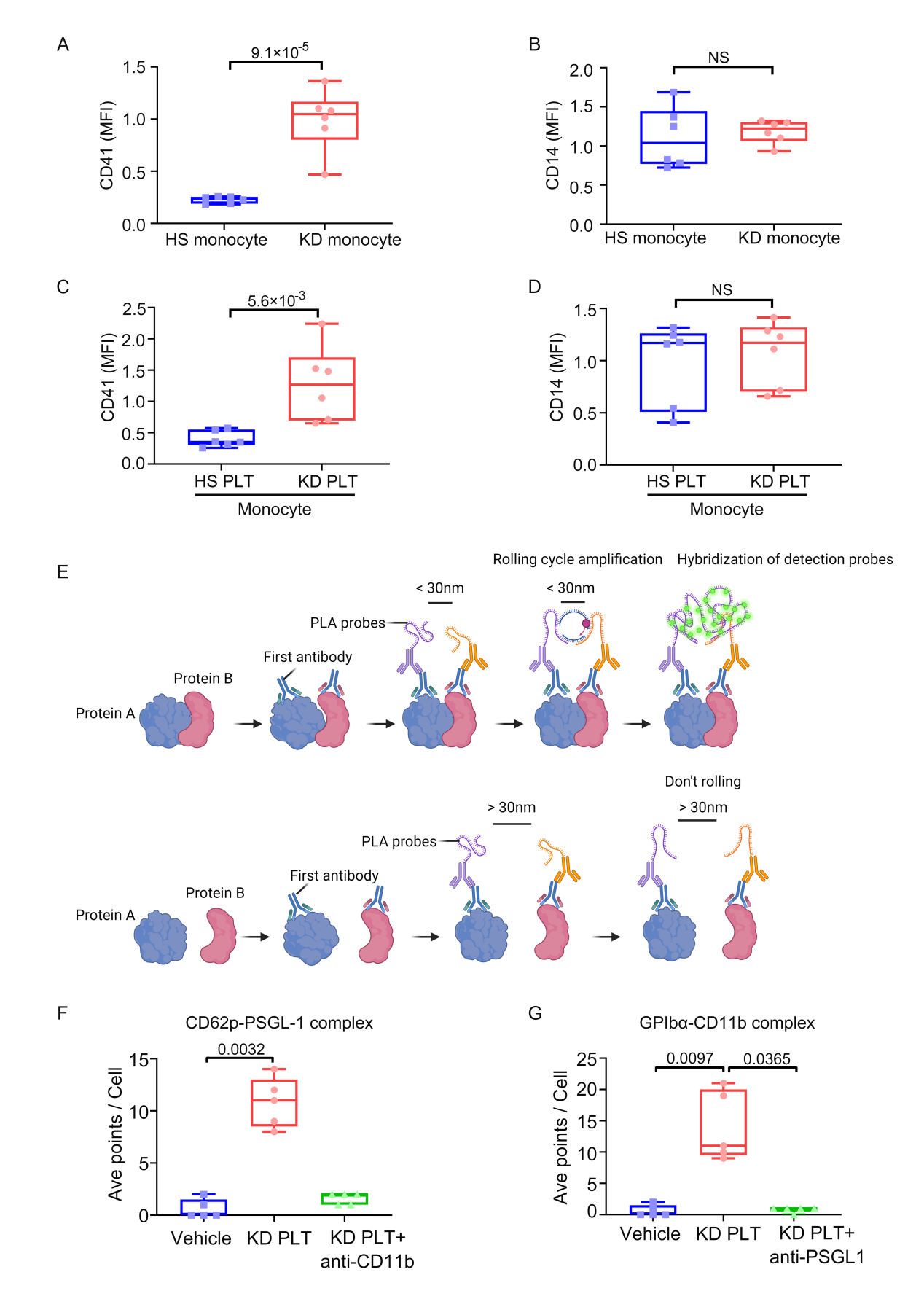


**Figure S2. Platelets are prone to aggregate with monocytes during acute KD.** (A-B) Quantification of CD41 (A) and CD14 (B) expression on the monocytes isolated from HS or patients with KD was shown (n=6). *P* values were calculated by Unpaired *t* test.

1. D) Quantification of CD41 (C) and CD14 (D) expression on monocytes after incubation with HS or KD platelets was shown (n=6). *P* values were calculated by Unpaired *t* test.
2. The schematic of a Proximity Ligation Assay (PLA) reaction.

(F-G) Monocytes were incubated with KD platelets in the presence of antibodies against CD11b or PSGL-1. PLA assays were performed to determine the direct interaction of partnership between CD62p / PSGL-1 (F), and GPIbα / CD11b (G) (n=5). Quantification of average points per cell in each group was determined by ImageJ, and subjected to statistical analysis for significance. Kruskal-Wallis test and Dunn's multiple comparisons test.

HS, healthy subject; KD, patients with Kawasaki disease; PLT, platelets; PLA, proximity ligation assays; NS, not significant.

**Figure S3**

**
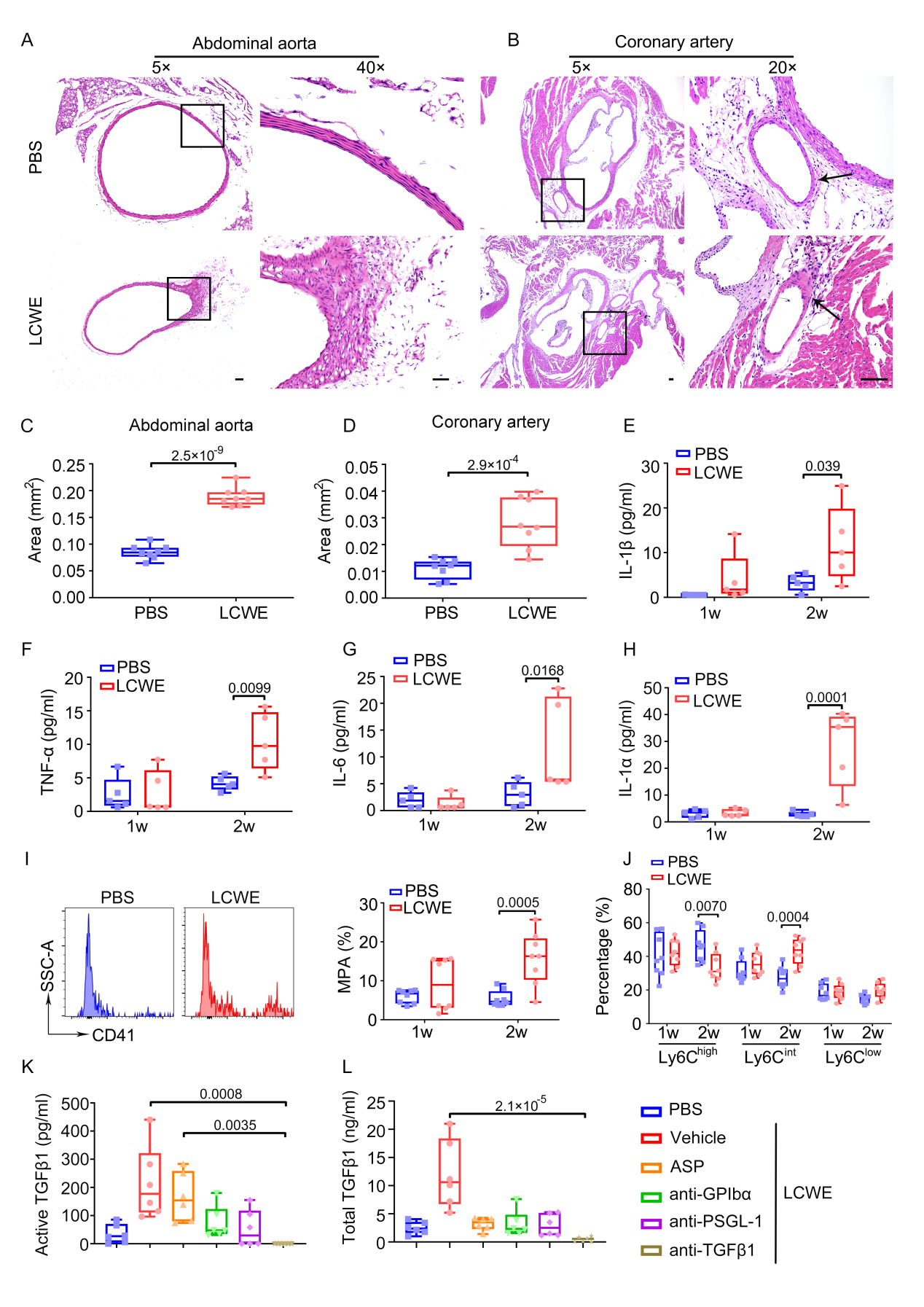
**

**Figure S3. Successful establishment of KD murine model with vasculitis**.

1. B) KD murine model was induced in C57BL/6 mice by intraperitoneal injection of LCWE. The frozen sections of the abdominal aorta and coronary artery were collected two weeks post-injection. Representative hematoxylin and eosin (H&E) staining of the abdominal aorta (A) and coronary artery (B) from mice injected with PBS (n=8) or LCWE were shown (n=8). Scale bar: 50μm.

(C-D) The areas of thickened media layer in the abdominal aorta (C) and coronary artery (D) from mice injected with PBS and LCWE were calculated by ImageJ software and subjected to statistical analysis for significance. *P* value was calculated by Unpaired *t* test.

(E-H) The levels of inflammation cytokine IL-1β (E), TNF-α (F), IL-6 (G), IL-1α (H) in plasma from PBS and LCWE-injected mice (n=5). *P* values were calculated by Two-way ANOVA and Sidak's multiple comparisons test.

(I) Flow cytometry analysis of MPA in peripheral blood from mice injected with PBS (n=8) or LCWE (n=8). Two-way ANOVA and Sidak's multiple comparisons test.

(J) Flow cytometry analysis of Ly6C^high^, Ly6C^int^, and Ly6C^low^ monocytes in peripheral blood from mice injected with PBS (n=8) or LCWE (n=8). Two-way ANOVA and Sidak's multiple comparisons test.

(K-L) The levels of active TGFβ1 (K) and total TGFβ1 (L) in plasma from PBS-injected mice (n=6), LCWE-injected mice (n=6), LCWE-injected mice followed by administration with ASP (n=6), anti-GPIbα (n=6), anti-PSGL-1 (n=6), anti-TGFβ1 (n=6). Mice injected with LCWE followed by administration of anti-TGFβ1 were used as a negative control. Kruskal-Wallis test and Dunn's multiple comparisons test.

LCWE, lactobacillus casei cell wall extract; ASP, aspirin.

**Figure S4**


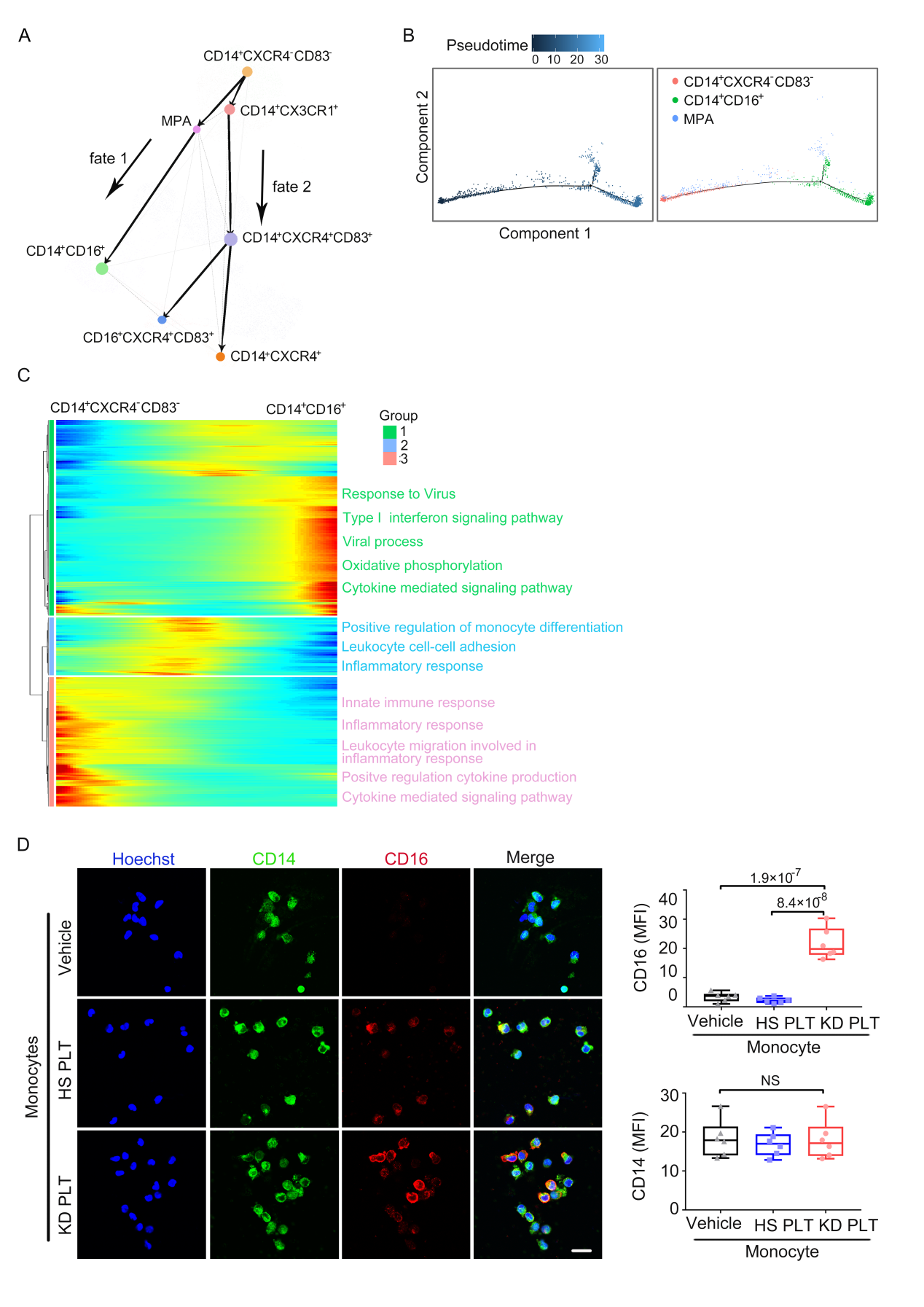


**Figure S4. KD platelets skew circulating monocytes towards a proinflammatory phenotype via the formation of “adhesion junctions”.**

1. PAGA graph representing the predicted path for monocytes from all subtypes.
2. Trajectory of monocytes in fate 1 along pseudotime in a two-dimensional space. Each point represents a single cell.
3. Heatmap showing the dynamic changes of gene expression along pseudotime. The differentially expressed genes were clustered hierarchically into three groups, and the representative enriched pathways of each group were shown.
4. Representative immunofluorescence and quantification of CD14 (green) and CD16 (red) in monocytes after co-culture with HS platelets and KD platelets for 16 hours (n=6). One-way ANOVA and Tukey's multiple comparisons test. Scale bar: 20μm.

HS, healthy subject; KD, patients with Kawasaki disease; PLT, platelets; NS, not significant.

**Figure S5**


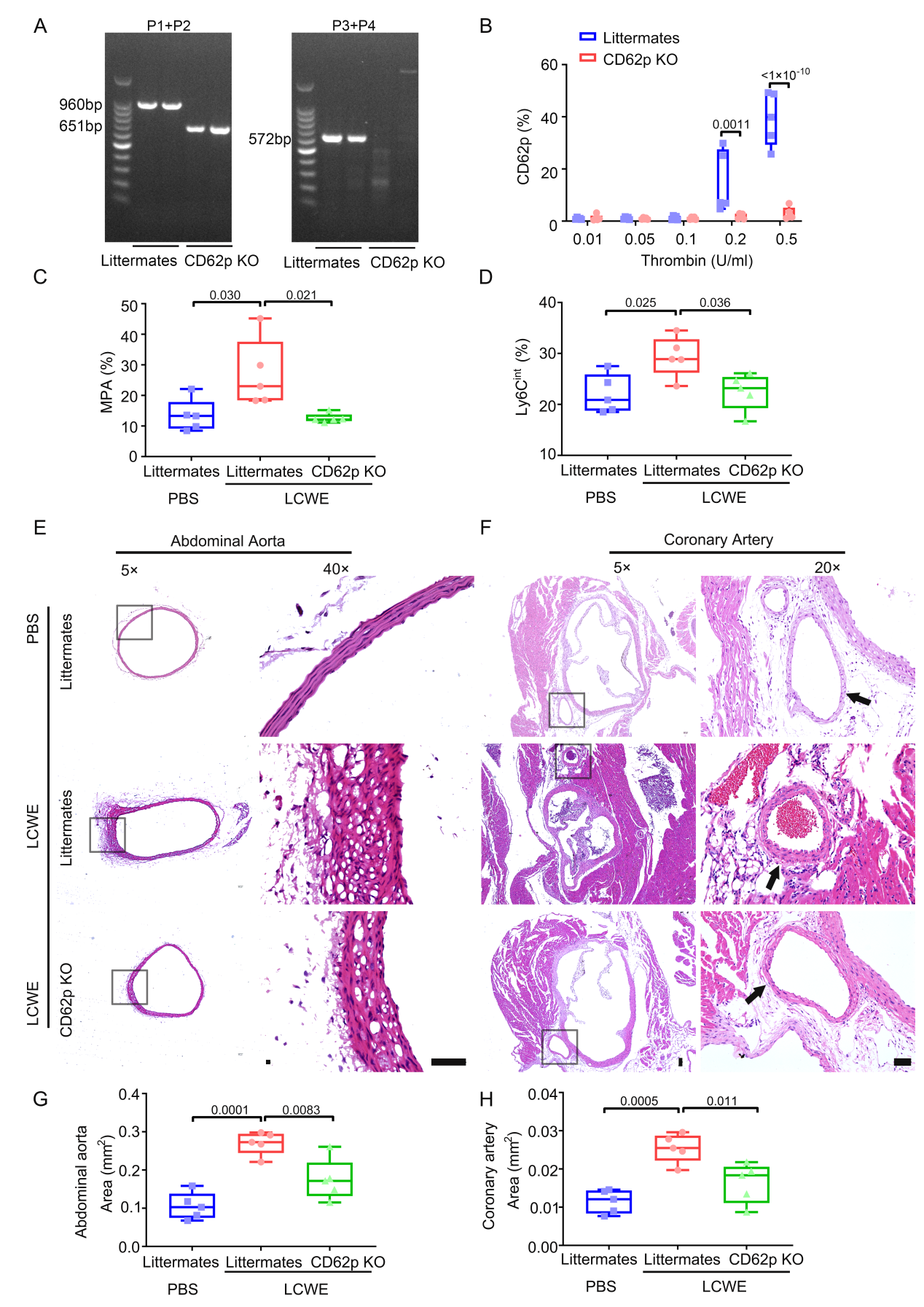


**Figure S5. Silencing of platelet CD62p alleviated LCWE-induced vasculopathy.** (A) Verification of CD62p excision in hematopoietic cells using genomic PCR.

1. Expression of CD62p using flow cytometry in platelets from littermates (n=5) and CD62p KO mice (n=5) after stimulation with thrombin at the concentration of 0.01, 0.05, 0.1, 0.2, 0.5 (U/ml), platelets from littermates were used as control. *P* values were calculated using Two-way ANOVA and Sidak's multiple comparisons test. (C-D) Bar plots showing MPA (C) and Ly6C^int^ monocytes (D) in peripheral blood from PBS-injected littermates (n=5), LCWE-injected littermates (n=5), and LCWE-injected CD62p KO mice (n=5). *P* values were calculated using One-way ANOVA and and Tukey's multiple comparisons test.

(E-F) KD murine model was induced by intraperitoneal injection of LCWE in littermates and CD62p KO mice. The frozen sections of the abdominal aorta and coronary artery were collected two weeks post-injection. Representative hematoxylin and eosin (H&E) staining of the abdominal aorta (E) and coronary artery (F) from mice injected with PBS (n=5) or LCWE were shown (n=5). Scale bar: 50μm.

(G-H) The areas of the thickened media layer in the abdominal aorta (G) and coronary artery (H) from each group were calculated by ImageJ software and subjected to statistical analysis for significance. One-way ANOVA and Tukey's multiple comparisons test.

LCWE, lactobacillus casei cell wall extract; KO, knockout; MPA, platelet-monocyte aggregate.

**Figure S6**

**
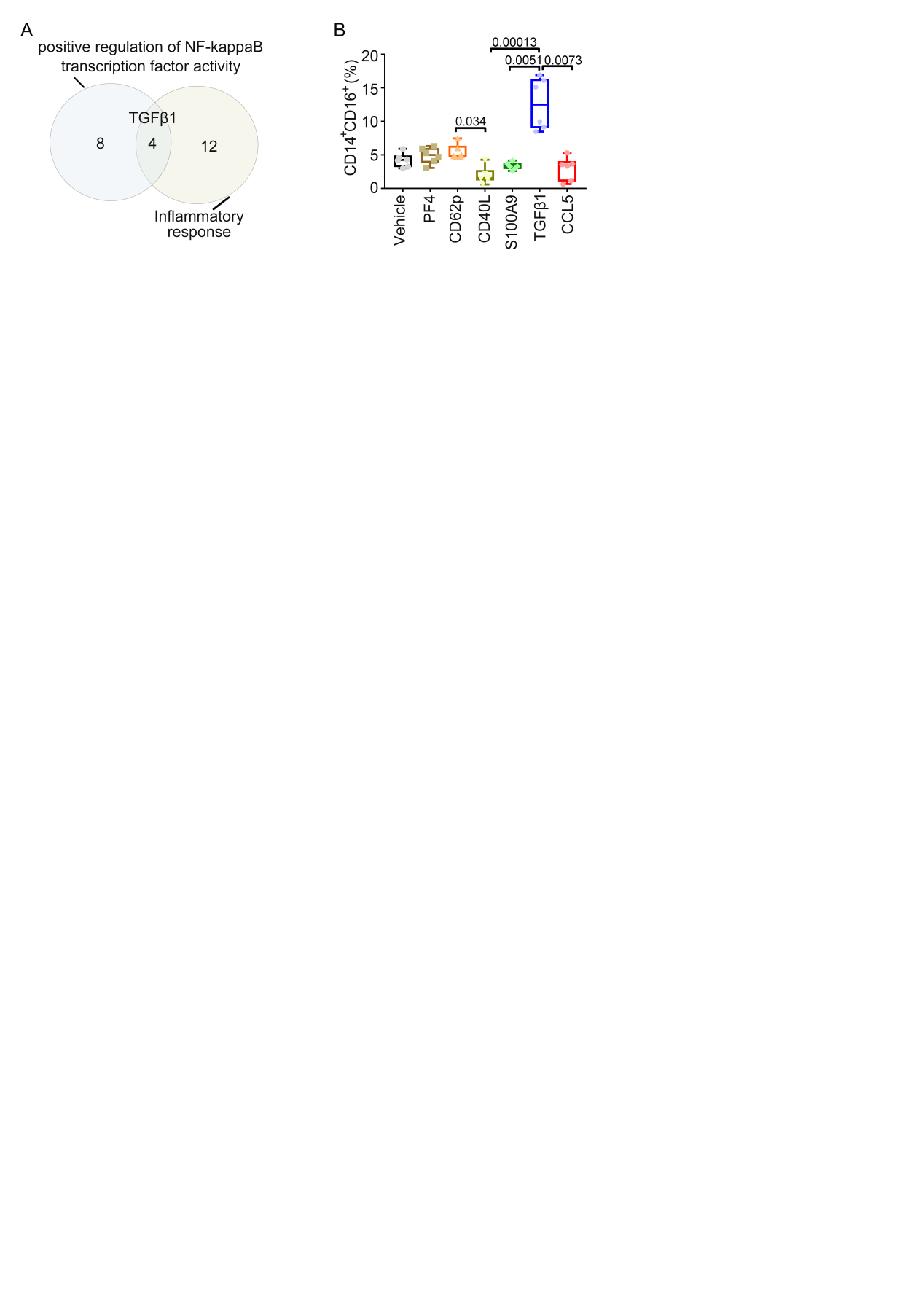
**

**Figure S6. “Adhesion junctions” between KD platelets and monocytes induced TGFβ release.**

1. Venn plot showing the intersection of genes involved in inflammation-related signaling pathways.
2. Flow cytometry analysis of the CD14^+^CD16^+^ monocyte after co-culture with the vehicle, or recombinant human cytokines including PF4 (0.2μg/ml), CD62p (0.5μg/ml), CD40L (1μg/ml), S100A9 (1μg/ml), TGFβ1 (10ng/ml), CCL5 (10ng/ml) (n=6). Kruskall-Wallis test and Dunn's multiple comparisons test.

HS, healthy subject; KD, patients with Kawasaki disease; PLT, platelets.

**Figure S7**


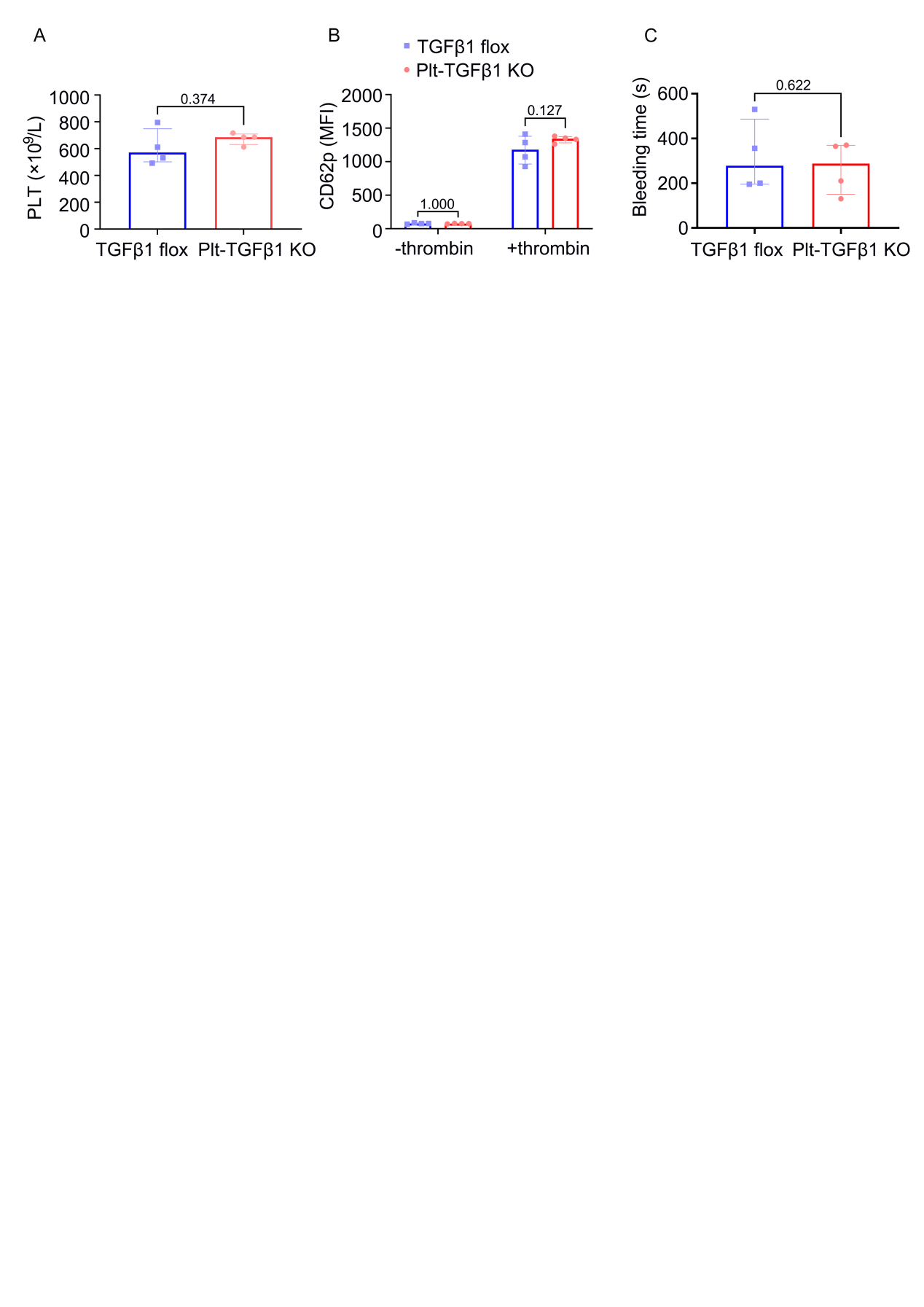


**Figure S7. Silencing of platelet TGFβ1 doesn’t affect platelet count, platelet activity, and tail bleeding time.**

1. Quantification of platelet count in Plt-TGFβ1 KO (TGFβ1^fl/fl^ PF4-Cre) mice (n=4) and TGFβ flox (TGFβ1^fl/fl^) mice (n=4). Unpaired *t* test.
2. Expression of CD62p using flow cytometry in platelets from Plt-TGFβ1 KO mice (n=4) and TGFβ1 flox mice (n=4) after stimulation with thrombin at the concentration of 0.5 (U/ml). *P* values were calculated using Two-way ANOVA and Sidak's multiple comparisons test.
3. Tail bleeding test was performed in Plt-TGFβ1 KO mice (n=4) and TGFβ1 flox mice (n =4). Each dot represents a mouse. Unpaired *t* test.

**Figure S8**

**
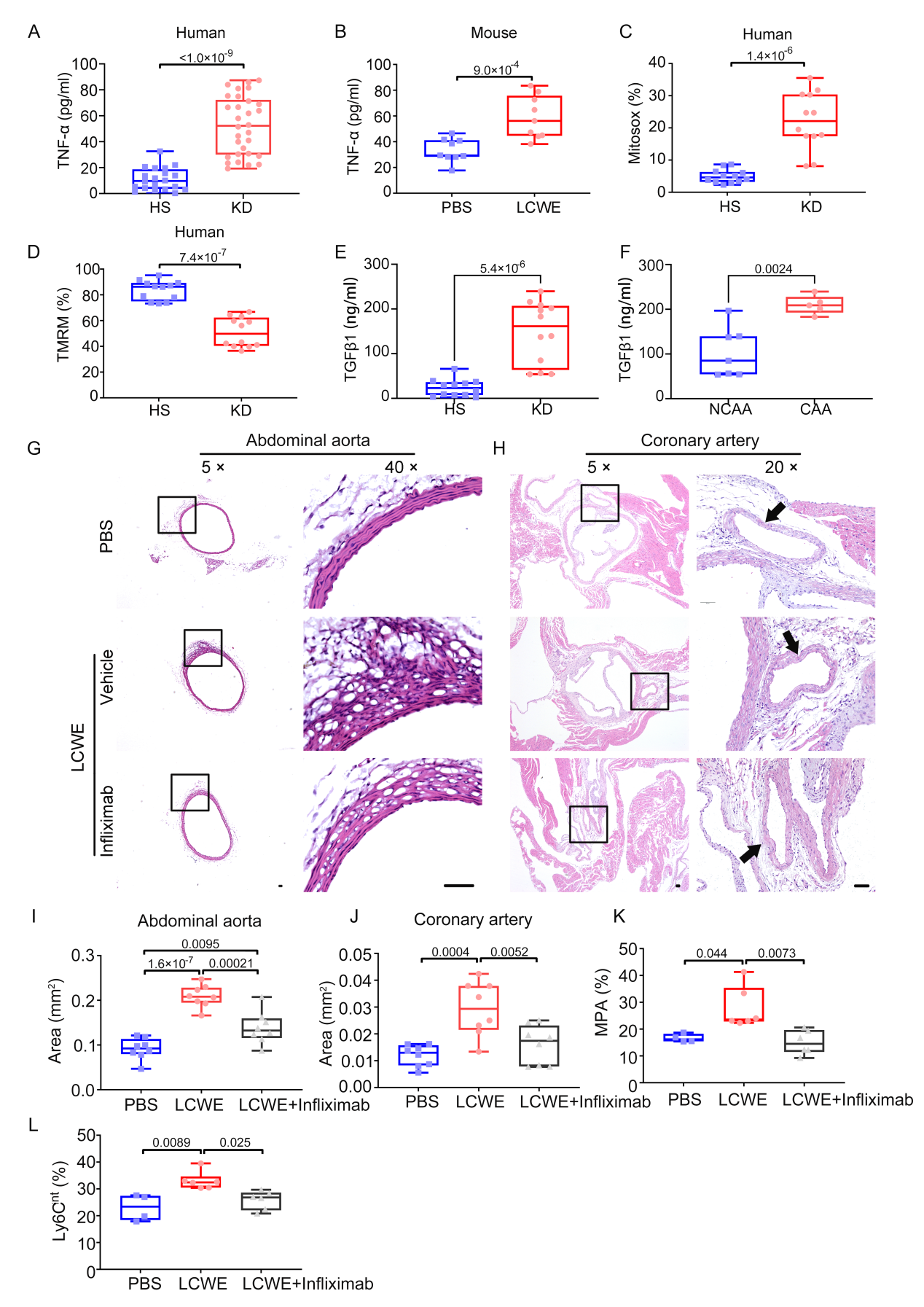
**

**Figure S8. TNF-α contributes to the induction of platelet hyperreactivity during acute KD.**

1. Level of TNF-α in plasma from participants with HS (n=20) and acute KD (n=30). Mann-Whitney test.
2. Level of TNF-α in plasma from LCWE-induced KD murine model (n=9) and PBS injected mice (n=9). Unpaired *t* test.
3. ROS production in platelets isolated from HS (n=12) and patients with acute KD (n=12) were monitored with MitoSOX. Unpaired *t* test.
4. Mitochondrial membrane potential in platelets isolated from HS (n=12) and patients with acute KD (n=12) were monitored with TMRM. Mann-Whitney test.
5. Level of Total TGFβ1 in plasma from HS (n=12) and patients with acute KD (n=12). Unpaired *t* test.
6. Level of Total TGFβ1 in plasma from patients with NCAA (n=7) and CAA (n=5) during acute KD. Unpaired *t* test.

(G-H) Mice were administrated with infliximab followed by LCWE injection. Representative H&E-stained sections of the abdominal aorta (G) and coronary artery (H) from PBS-injected mice (n=8), LCWE-injected mice (n=8), LCWE-injected mice followed by administration with infliximab (n=8), were shown. Scale bar: 50μm.

(I-J) The areas of thickened media layer in abdominal aorta (I) and coronary artery (J) from each group were calculated by ImageJ software and subjected to statistical analysis for significance. *P* value was calculated by One-way ANOVA and Tukey's multiple comparisons test.

(K-L) Flow cytometry analysis of MPA (K) and Ly6C^int^ monocytes (L) in peripheral blood from PBS-injected mice (n=4), LCWE-injected mice (n=6), LCWE-injected mice followed by administration with infliximab (n=6) was shown (Kruskall-Wallis test and Dunn's multiple comparisons test).

LCWE, lactobacillus casei cell wall extract; MPA, platelet-monocyte aggregate.
